# The psychological legacy of past obesity and early mortality: evidence from two longitudinal studies

**DOI:** 10.1186/s12916-023-03148-3

**Published:** 2023-11-16

**Authors:** I Gusti Ngurah Edi Putra, Michael Daly, Angelina Sutin, Andrew Steptoe, Eric Robinson

**Affiliations:** 1 Department of Psychology, Institute of Population Health, University of Liverpool, Eleanor Rathbone Building, Bedford Street South, Liverpool, L69 7ZA UK; 2 Department of Psychology, Maynooth University, Maynooth, Ireland; 3 Department of Behavioral Sciences and Social Medicine, Florida State University College of Medicine, Tallahassee, USA; 4 Department of Behavioural Science and Health, Institute of Epidemiology and Health Care, Faculty of Population Health Sciences, University College London, London, UK

**Keywords:** Obesity, Mortality, Weight scarring, Psychological well-being, Psychological distress, Depressive symptoms

## Abstract

**Background:**

We test a novel ‘weight scarring’ hypothesis which suggests that past obesity is associated with impairments in current psychological well-being and this increases risk of negative physical health outcomes associated with obesity. Across two nationally representative studies, we tested whether past obesity is associated with current psychological outcomes and whether these psychological outcomes explain the association between past obesity and subsequent early mortality.

**Methods:**

Data were from the National Health and Nutrition Examination Survey (NHANES) (*n* = 29,047) and the Health and Retirement Study (HRS) (*n* = 11,998). Past obesity was defined based on maximum lifetime weight in NHANES and the highest weight from past study waves in the HRS. Across both studies, current depressive symptoms were analysed. A set of 10 additional well-being measures were combined to produce an ‘index of impaired well-being’ in HRS. Subsequent all-cause mortality was examined using National Deaths Index records in NHANES and household interviews in HRS. Linear or logistic regression, Cox proportional hazard regression, and causal mediation models were used.

**Results:**

We found that past obesity was associated with greater current depressive symptoms after controlling for current weight status and in analyses limited to those who were no longer classified as having obesity in NHANES (*β* = 0.17; 95% CI: 0.13, 0.22) and HRS (*β* = 0.20; 95% CI: 0.08, 0.31). In HRS, past obesity was also associated with a range of current negative psychological outcomes, including an index of impaired psychological well-being (*β* = 0.16; 95% CI: 0.05, 0.27). Past obesity was associated with a higher risk of early mortality in both NHANES and HRS (HR = 1.31; 95% CI: 1.16, 1.48 and HR = 1.34; 95% CI: 1.20, 1.50, respectively). Depressive symptoms explained 6% (95% CI: 0.01, 0.10) and 5% (95% CI: 0.01, 0.09) of the association between past obesity and premature mortality in NHANES and HRS, respectively. Impaired psychological well-being partly mediated the association between past obesity and premature mortality by 10% (95% CI: 0.04, 0.16) in HRS.

**Conclusions:**

Our findings suggest that there may be a psychological legacy of past obesity that is associated with raised mortality risk. Ensuring people with obesity receive psychological support even after experiencing weight loss may be important.

**Supplementary Information:**

The online version contains supplementary material available at 10.1186/s12916-023-03148-3.

## Background

Obesity has been considered a multifactorial disease, resulting from complex interactions between individual and environmental factors [1, 2]. Current evidence indicates that individual factors, including socioeconomic status (e.g. education, income) [3, 4], health-related behaviour (e.g. physical activity, diet) [5, 6], and environmental factors, including food marketing [7], built environments [8], and obesity-related policies (e.g. menu labelling) [9], can play an important role in shaping the prevalence of obesity. The global prevalence of obesity has nearly tripled in the last four decades (1975–2016) and is now a major public health crisis [10]. Obesity is associated with a significant disease burden [11] and is estimated to reduce life expectancy by up to 14 years [12]. There is also a significant psychological burden of obesity, as current obesity is associated with an increased risk of depression and other psychiatric illnesses [13, 14]. To date, research on obesity and mental health has largely taken a short-term perspective and assumed that weight loss should largely ameliorate the adverse psychological consequences of obesity [15]. The present work takes a different approach and tests a ‘weight scarring’ hypothesis which proposes that past obesity is associated with worse current psychological outcomes even after weight loss and these harmful psychological outcomes are associated with subsequent adverse physical health.

It is well established that the experience of stigma and weight-based discrimination is a chronic source of psychological distress among people living with obesity [16]. Weight stigma is so ubiquitous that people living with obesity often internalise the negative stereotypes and attitudes associated with obesity [17, 18]. Qualitative research suggests that even among those who have lost weight to the point of no longer being classed as having obesity, many fear being stigmatised and may also be treated differently due to having previously had obesity [19, 20]. Furthermore, obesity and depression share similar biological pathways, and by raising inflammation in the body [21], a history of obesity may be associated with long-lasting biological changes that impair psychological well-being and are not fully reversible even after weight loss [22]. Consistent with work challenging set-point models of psychological well-being [23, 24], we propose that having lived with obesity may act as a significant life event and large numbers of people are not fully able to recover from its psychological impact. In the present research, we therefore test for the first time whether past obesity appears to be *psychologically ‘scarring’* and whether the psychological outcomes associated with obesity may increase the risk of premature mortality.

Although a small amount of methodologically limited research has examined weight during childhood and depression in later life [25], there is a dearth of evidence on the association between previous obesity and psychological well-being after weight loss. If past obesity is associated with psychological well-being, then this may also have substantial implications for future physical health. There is consistent evidence that compromised psychological well-being, such as depression [26], anxiety [27], and loneliness [28] harm physical health and are associated with increased mortality risk, which may occur indirectly via health behaviour [29–31] or directly through changes in physiological conditions on immune, metabolic and cardiovascular health [32, 33]. In the present research, we therefore test for the first time whether the psychological legacy associated with past obesity (i.e. impaired well-being) in part explains why obesity is a risk factor for premature mortality.

The present research aimed to (1) quantify the association between past obesity and a range of psychological outcomes and (2) examine the extent to which psychological well-being explains why past obesity is associated with the increased risk of mortality. To address these aims, we build on previous work that models the association between obesity and mortality risk by accounting for both current and past obesity (i.e. highest previous body mass index—BMI) because examining current BMI alone has been shown to provide an incomplete picture of the association between obesity in adult life and early mortality [34–36]. In the present study, we used two large representative US studies. The US has one of the highest national prevalences of obesity the world [37], which is more than 40% in adults aged 20 years and over [38]. We first test our hypotheses in the National Health and Nutrition Examination Survey (NHANES) and then extend and examine generalizability of findings to the Health and Retirement Study (HRS).

## Methods

### National Health and Nutrition Examination Survey (NHANES)

**Fig. 1:**
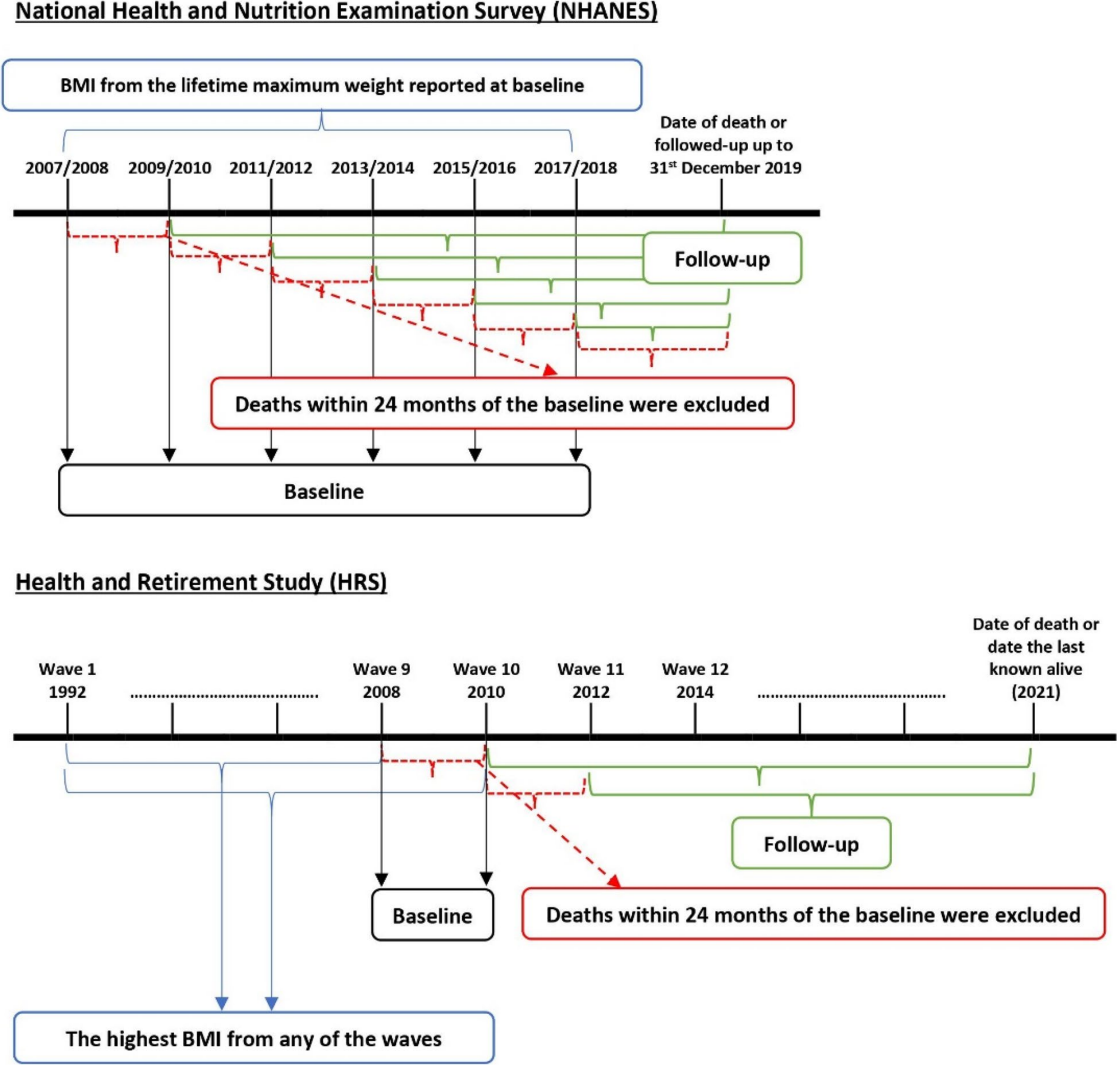
Diagram of the study design. For both NHANES and HRS, psychological outcomes and sociodemographic covariates at baseline were examined. In HRS, a measure of depressive symptoms was available in every wave, and therefore, pre-baseline depressive symptoms prior to when obesity developed were controlled for to estimate the effect of past obesity on current (baseline) depressive symptoms

We used data from NHANES, a nationally representative survey of the US non-institutionalised resident population. NHANES recruits a representative sample of over 5000 adults every 2 years using multistage probability random sampling. Participants completed a face-to-face interview and physical examination [39]. The current study drew on data from the NHANES weight history assessment, physical examination of body weight, assessment of depressive symptoms, and follow-up tracking of mortality. Participants from six rounds of NHANES from 2007/2008 to 2017/2018 were combined. The time of NHANES assessment was treated as ‘baseline’ and linked to past obesity and subsequent mortality assessed via records available from the National Death Index (NDI) available up to 31 December 2019 [40] (Fig. 1). To avoid reverse causality (i.e. cause and effect are reversed in which the presence of illness leads to reduced BMI and early mortality), participants with survival time ≤ 24 months from the baseline interview date were excluded [41]. We also omitted participants with underweight (*n* = 534), making the final sample size of 29,047. The National Center for Health Statistics Ethics Review Board provided ethics approval for NHANES.

### Health and Retirement Study (HRS)

We replicated and extended the findings from NHANES using HRS. HRS has a wider range of psychological outcomes and retrospective longitudinal data of weight records. HRS is a nationally representative longitudinal study of approximately 20,000 US non-institutionalised older adults aged ≥ 50 years and their spouse regardless of age (all participants and spouses under 50 were excluded). HRS participants were selected using multistage area probability sampling design. HRS began in 1992 (wave 1) and participants are reinterviewed every 2 years (waves 2 onwards). A mixed-mode design began in 2006 (wave 8) to expand HRS content by collecting physical, biological, and psychological outcomes through an enhanced face-to-face interview (EFTF). Half of the HRS participants completed EFTF in 2006 (wave 8), and the other half participated in 2008 (wave 9), which is then repeated every 4 years [42]. The ‘baseline’ for this present study was selected at waves 9 and 10 (2008 and 2010) since all psychological outcomes were consistently assessed using the same tools (Fig. 1). Similar to NHANES, we removed participants with survival time ≤ 24 months and always underweight (BMI < 18.5) (*n* = 30), making the final sample size of 11,998. Ethics approval for HRS was obtained from the University of Michigan Institutional Review Board.

### Obesity status

In NHANES, the highest past BMI (kg/m^2^) was determined from greatest weight and height self-reported in the weight history assessment (e.g. as in [35]). Current BMI was assessed using staff-assessed height and weight in every round of the study. Trained staff assessed weight (in pounds) using digital weight scale and height (in inches) using stadiometer as part of the NHANES physical examination. In the HRS, we defined past BMI as the maximum BMI based on self-reported weight and height in any biennial assessments before and up to baseline (e.g. as in [36]). In HRS, objective measures of weight and height were collected as part of the EFTF (waves 8 onwards). Trained interviewers measured participants’ weight (in pounds) using a Healthometer 830KL scale and height (in inches) using a tape measure. Across both studies, the highest past BMI was classified as non-obesity (BMI < 30) and obesity (BMI ≥ 30), and objective BMI measured at baseline (current BMI) was controlled for in the analyses.

### Psychological outcomes

In NHANES, depressive symptoms were the only available psychological outcome across recent NHANES surveys. HRS collected numerous psychological outcomes, and we examined all psychological outcomes that we reasoned might explain the prospective association between obesity and mortality. Sixteen psychological outcomes were included (depressive symptoms, life satisfaction, loneliness, weight stigma, social support, social strain, positive affect, negative affect, purpose in life, anxiety, hopelessness, optimism, pessimism, cynical hostility, personal constraint, mastery). Psychological outcomes were in the form of continuous scores, except weight stigma which was a dichotomous variable (yes; no) (see Additional file 1 for full information on how psychological outcomes were defined). All individual continuous psychological outcomes were transformed into *z*-scores to allow comparison across different metrics.

In HRS, we also aimed to test the collective mediation by psychological outcomes on the association between past obesity and the risk of mortality. However, testing all individual psychological outcomes together in a multiple mediation analysis would result in multicollinearity due to moderately to high correlations between variables (see Additional file 2: Table S1 for full information). We therefore used exploratory factor analysis (EFA) to identify if a collection of psychological outcomes formed an underlying latent construct. We used item loading cut-off values of 0.55 (good item loading) to retain psychological outcomes to develop a composite index (e.g. as in [43]) (see Additional file 1 for full information). Out of 16 psychological outcomes included in the EFA, 10 variables were loaded onto a single factor (depressive symptoms, life satisfaction, loneliness, positive affect, negative affect, purpose in life, anxiety, hopelessness, pessimism, and personal constraint) that we termed ‘impaired psychological well-being’. All retained psychological outcomes (*n* = 10) were coded so that higher scores indicated greater impairment, *z*-score standardised, averaged, and re-standardised to generate an index with a mean of 0 and a standard deviation (SD) of 1 (see Additional file 1 for full information on convergent validity and internal consistency of the index).

### Mortality

We linked NHANES surveys to mortality records available from NDI through 31 December 2019 [40]. Survival time (in months) in NHANES was calculated from the interview day (month and year) at baseline (2007/2008–2017/2018) to the death date or up to 31 December 2019 for participants who were alive. Because mortality records from NDI are no longer available in the HRS, we used the most up-to-date mortality status from a household proxy report (interviews or personal communications with family members). This information was available up to early 2021 via the latest release of the cross-wave tracker file (June 2022) [44]. Survival time in HRS was computed in months from the interview day (month and year) at baseline (waves 9 and 10) to the last day when participants were known to be alive (e.g. last interview date) or dead (e.g. as in [45]).

### Covariates

Baseline sociodemographic covariates were controlled for in both samples, including age (in years), sex (male; female), ethnicity (non-White; White), marital status (no married; married or cohabitating), number of years of education, and paid employment status (no; yes). We also controlled for quantiles of household income in NHANES and quintiles of total household wealth in HRS. All analyses also controlled for current objective BMI and current BMI-squared to account for the non-linear relationship between BMI and psychological outcomes. As psychological outcomes (e.g. depressive symptoms) may be associated with lifestyle related health behaviours [46] and chronic conditions [47], in sensitivity analyses we controlled for available health behaviours (smoking, physical activity, drinking behaviour) and chronic conditions (hypertension, heart disease, stroke, diabetes, arthritis, cancer) in the mediation models (see the ‘Sensitivity analyses’ section).

### Data analysis

To compensate for unequal probability of being selected as a participant, we incorporated sample weights in the analysis across studies to make the findings representative to the population. We first tested whether past obesity status (obesity vs. non-obesity) was associated with worse psychological outcomes at baseline. Linear or logistic regression was used to evaluate whether past obesity status was associated with all psychological outcomes at baseline, adjusting for covariates and current objective BMI and BMI-squared. This analysis was repeated on participants who did not have obesity (BMI < 30) at baseline. We then used Cox proportional hazard regression models to examine the prospective association between past obesity, psychological outcomes, and the risk of mortality with adjustment for covariates and current weight status. The risk of mortality was presented as hazard ratio (HR) along with 95% confidence intervals (CI) and *p*-value.

We used the STATA command, med4way, to conduct causal mediation analyses with survival outcomes [48]. This mediation analysis decomposes the total effect into four components: (1) controlled direct effect (‘not explained by either the mediator or exposure-mediator interaction; explained only by the exposure’), (2) reference interaction (‘explained only by the interaction between the exposure and the mediator’), (3) mediated interaction (‘explained by both interaction and mediation or mediated interaction’), and (4) pure indirect effect (explained only by mediation) [48, 49]. Mediated interaction represents the additional interaction that is only apparent when the exposure is associated with the mediator. Collectively, both proportions due to mediated interaction and pure indirect effect represent the overall proportion due to mediation or the proportion mediated [49]. Med4way only allows for single mediation models that were developed by fitting together two regression models of the exposure on the mediator and the exposure on the outcome, adjusted for and in interaction with the potential mediator. Linear or logistic regression was selected for a model of the exposure on the mediator (logistic regression for a model of obesity on weight stigma only). However, Cox regression was not used to estimate the association between the exposure and the outcome since it might yield biased estimates in mediation analyses of non-rare outcomes as was informed by previous studies [50–52]. We used an accelerated failure time (AFT) model with a Weibull distribution to estimate the effect of obesity on mortality (e.g. as in [52]). AFT analyses produce survival time ratios (TRs) as disease-free time. Contrary to Cox regression where HR > 1 indicates increased risk, TR > 1 from ART models indicates longer disease-free time. In this present study, the association between past obesity and mortality at mean levels of the mediator were tested [52, 53]. Separate single mediation models were carried out to test mediation by individual psychological outcomes and an index of impaired psychological well-being. Findings from mediation analyses using med4way were presented as total effect relative risk ratio or TR, proportions of controlled direct effect, reference interaction, mediated interaction, and pure indirect effect. Overall proportions due to mediation, interaction, and proportion eliminated along with 95% CI and *p*-value were also presented.

In NHANES, missing values were present for marital status (4.95% of participants) and household income (9.46% of participants). Dummy variables were included to account for differences in depressive symptom levels between those with/without data on marital status and household income. HRS utilised data from several waves of the study and a large number of psychological outcomes and therefore required a more complex strategy to account for attrition and missing data. In HRS, 1782 out of 11,198 observations (weighted percentage = 14.85%) were missing data on at least one of the sociodemographic covariates, weight status, or psychological outcomes. Participants who were older, non-White, not currently married or cohabitating, not working, from lower wealth quantiles, and had fewer years of education were more likely to be excluded from the analytic sample due to missing values. We adjusted baseline sample weights to account for these missing observations [54, 55]. Using a logistic regression model, all sociodemographic covariates were used to estimate the probability or propensity scores of being retained at follow-up or included in the analytic sample. New sample weights were calculated as the inverse probability of being retained and then combined with baseline weights. These weights were applied in all the analyses to compensate for the differences in participants’ characteristics between those who were retained (analytical sample) and who were not, as well as non-response characteristics at baseline. For Cox proportional hazard regression and mediation models, we included these weights when data were converted for survival analysis.

### Sensitivity analyses

In HRS, some participants entered the cohort for the first time as a replenished sample in wave 10 (part of the baseline) (*n* = 1039). In addition, other participants had missing information on their self-reported BMI at all waves before the baseline (*n* = 400). Therefore, these participants would have their highest past BMI the same as their current or baseline BMI. We conducted sensitivity analyses in HRS by excluding participants whose weight history was only available at baseline (*n* = 1439). Sensitivity analyses were also conducted to examine whether the association between past obesity and current psychological outcomes remained when psychological outcomes collected prior to past obesity were accounted for. Given depressive symptoms were the only psychological outcome collected across the HRS waves, we limited the analyses on current depressive symptoms, controlling past depressive symptoms before developing obesity. Past depressive symptoms (i.e. pre-baseline) were extracted from the first wave of HRS where participants did not have obesity. Participants were excluded if they had obesity in all HRS waves, weight history or weight status available at baseline only, and/or no pre-baseline measure of depressive symptoms. In both studies, we also examined the extent to which mediation by psychological measures on the association between past obesity and increased risk of mortality persisted when health behaviours (smoking, physical activity, drinking) and chronic conditions (hypertension, heart disease, stroke, diabetes, arthritis, cancer) were controlled for in the mediation models.

## Results

### Characteristics of participants

Table 1 presents baseline characteristics of the participants across studies. Equivalent numbers of females and males were sampled, and most participants were White (NHANES: 67.4%, HRS: 85.3%). In NHANES, 47.9% of the sample self-reported past obesity, and 37.9% currently had obesity. In HRS, 42.7% of the participants reported past obesity, and 42.4% currently had obesity. In NHANES, 25.1% of those with past obesity were no longer classed as having obesity at baseline. In HRS, 14.0% of those with past obesity no longer had obesity at baseline. In NHANES, out of 29,047 participants (198,108 person-years), 1985 (6.8%) died during follow-up. In HRS, there were 11,998 participants (109,531 person-years), and 3501 (23.9%) died during follow-up.

**Table 1:** Baseline characteristics of participants

**Variables**	**NHANES**		**HRS**	
	***n* = 29,047**	**%**^**a**^	***n* = 11,998**	**%**^**a**^
Age				
Min, max		18; 80		50; 107
Mean (SD)		46.52 (17.27)		65.17 (10.09)
Sex				
Female	14,405	49.26	7093	54.83
Male	14,642	50.74	4905	45.17
Ethnicity				
Non-White	12,057	32.59	2311	14.67
White	16,990	67.41	9617	85.33
Highest past BMI				
Non-obesity	14,886	52.12	6779	57.30
Obesity	14,181	47.88	5181	42.70
Current/baseline BMI				
Non-obesity	17,799	62.07	6554	57.65
Obesity	11,248	37.93	4774	42.35
Death status				
No	27,062	93.17	8497	76.11
Yes	1985	6.83	3501	23.89

*n* Maximum analytical sample size, *min* Minimum value, *max* Maximum value, *SD* Standard deviation, *%* Percentage, *BMI* Body mass index

^a^Weighted by respondent baseline weights

### Association between past obesity and psychological outcomes

Table 2 presents the associations between past obesity status and baseline psychological outcomes with adjustment for basic sociodemographic covariates and baseline BMI and BMI-squared for all participants (model 1) and among participants without current obesity (model 2). In both studies, past obesity was associated with more depressive symptoms (NHANES: *β* = 0.17; 95% CI: 0.13, 0.21; HRS: *β* = 0.12, 95% CI: 0.05, 0.18), and in HRS, past obesity was associated with worse psychological well-being across a range of available indicators including a combined index of impaired psychological well-being (*β* = 0.15, 95% CI: 0.09, 0.21) (model 1). Importantly, the association between past obesity and depressive symptoms (NHANES: *β* = 0.17, 95% CI: 0.13, 0.22; HRS: *β* = 0.20, 95% CI: 0.08, 0.31) and the majority of other psychological outcomes in the HRS remained when the analysis was limited to participants without current obesity (model 2). Thus, past obesity was associated with current psychological well-being irrespective of current BMI or obesity status. To further test the ‘scarring’ hypothesis, in HRS, we were able to examine if the above findings on depressive symptoms remained when controlling for depressive symptoms assessed prior to when participants developed past obesity. We found that the magnitude of the association between past obesity and current depressive symptoms was slightly reduced but remained statistically significant (*β* = 0.11; 95% CI: 0.04, 0.18 when including all participants and *β* = 0.18; 95% CI: 0.04, 0.31 when excluding participants with current obesity) after controlling for levels of depressive symptoms before developing obesity (Additional file 2: Table S2). In other words, past obesity was associated with current depressive symptoms independent of earlier life depressive symptoms prior to developing obesity.

**Table 2:** Associations between past obesity (vs no past obesity) and current psychological outcomes

**Psychological outcomes**	**Past obesity status (obesity vs. non-obesity)**					
	***n***	**Model 1**		***n***	**Model 2**	
		**Estimate (95% CI)**	***p*-value**		**Estimate (95% CI)**	***p*-value**
***National Health and Nutrition Examination Survey (NHANES)***						
Depressive symptoms (PHQ-9)	29,047	0.17 (0.13, 0.21)	< 0.001	17,799	0.17 (0.13, 0.22)	< 0.001
***Health and Retirement Study (HRS)***						
Depressive symptoms (CES-D-8)	11,195	0.12 (0.05, 0.18)	< 0.001	6482	0.20 (0.08, 0.31)	0.001
Life satisfaction	11,072	−0.10 (−0.17, −0.04)	0.001	6410	−0.11 (−0.21, −0.01)	0.040
Loneliness	11,010	0.11 (0.04, 0.17)	0.001	6374	0.12 (0.02, 0.23)	0.022
Social support	11,152	−0.07 (−0.14, −0.01)	0.025	6454	−0.06 (−0.17, 0.04)	0.227
Social strain	11,144	0.11 (0.05, 0.18)	< 0.001	6450	0.14 (0.04, 0.24)	0.007
Positive affect	11,029	−0.13 (−0.19, −0.06)	< 0.001	6383	−0.11 (−0.21, 0.00)	0.053
Negative affect	11,035	0.12 (0.05, 0.19)	0.001	6387	0.17 (0.06, 0.29)	0.003
Purpose in life	10,968	−0.10 (−0.16, −0.03)	0.003	6328	−0.04 (−0.14, 0.06)	0.451
Anxiety	11,026	0.14 (0.08, 0.21)	< 0.001	6371	0.22 (0.10, 0.34)	< 0.001
Hopelessness	11,082	0.07 (0.01, 0.13)	0.024	6410	0.03 (−0.07, 0.13)	0.602
Optimism	11,020	−0.06 (−0.12, 0.01)	0.101	6375	−0.06 (−0.17, 0.05)	0.314
Pessimism	11,013	0.06 (−0.00, 0.12)	0.059	6371	0.03 (−0.07, 0.12)	0.608
Cynical hostility	10,816	0.03 (−0.03, 0.09)	0.336	6263	−0.01 (−0.11, 0.08)	0.813
Personal constrain	11,064	0.08 (0.01, 0.14)	0.017	6407	0.09 (−0.02, 0.19)	0.094
Mastery	11,067	−0.10 (−0.16, −0.03)	0.003	6411	−0.13 (−0.24, −0.03)	0.012
Weight stigma *(ref: no)*	11,086	3.53 (2.65, 4.70)	< 0.001	6407	3.60 (2.16, 5.99)	< 0.001
Index of impaired psychological well-being	11,143	0.15 (0.09, 0.21)	< 0.001	6450	0.16 (0.05, 0.27)	0.004

Model 1 included all eligible participants, and model 2 included only participants with no obesity at baseline

Separate regression models were developed for each psychological adjusting for age, sex, ethnicity, marital status, education, working status, household income (NHANES) or household wealth (HRS), current objective BMI and BMI-squared, and study wave (NHANES)

Findings for the adjusted associations between past obesity status and psychological outcomes are presented as regression coefficients (*β*), except for weight stigma presented as odds ratio (OR)

All continuous psychological outcomes were in the form of *z*-score (mean = 0; SD = 1)

Index of impaired psychological well-being was developed by re-standardising the average standardised scores of 10 psychological outcomes (depressive symptoms, life satisfaction, loneliness, positive affect, negative affect, purpose in life, anxiety, hopelessness, pessimism, and personal constraint)

*n* Analytical sample size, *CI* Confidence interval, *ref* Reference group, *PHQ-9* Patient Health Questionnaire (9 items), *CES-D-8* Center for Epidemiologic Studies Depression Scale (8 items)

### Associations between past obesity, psychological outcomes, and mortality

**Fig. 2:**
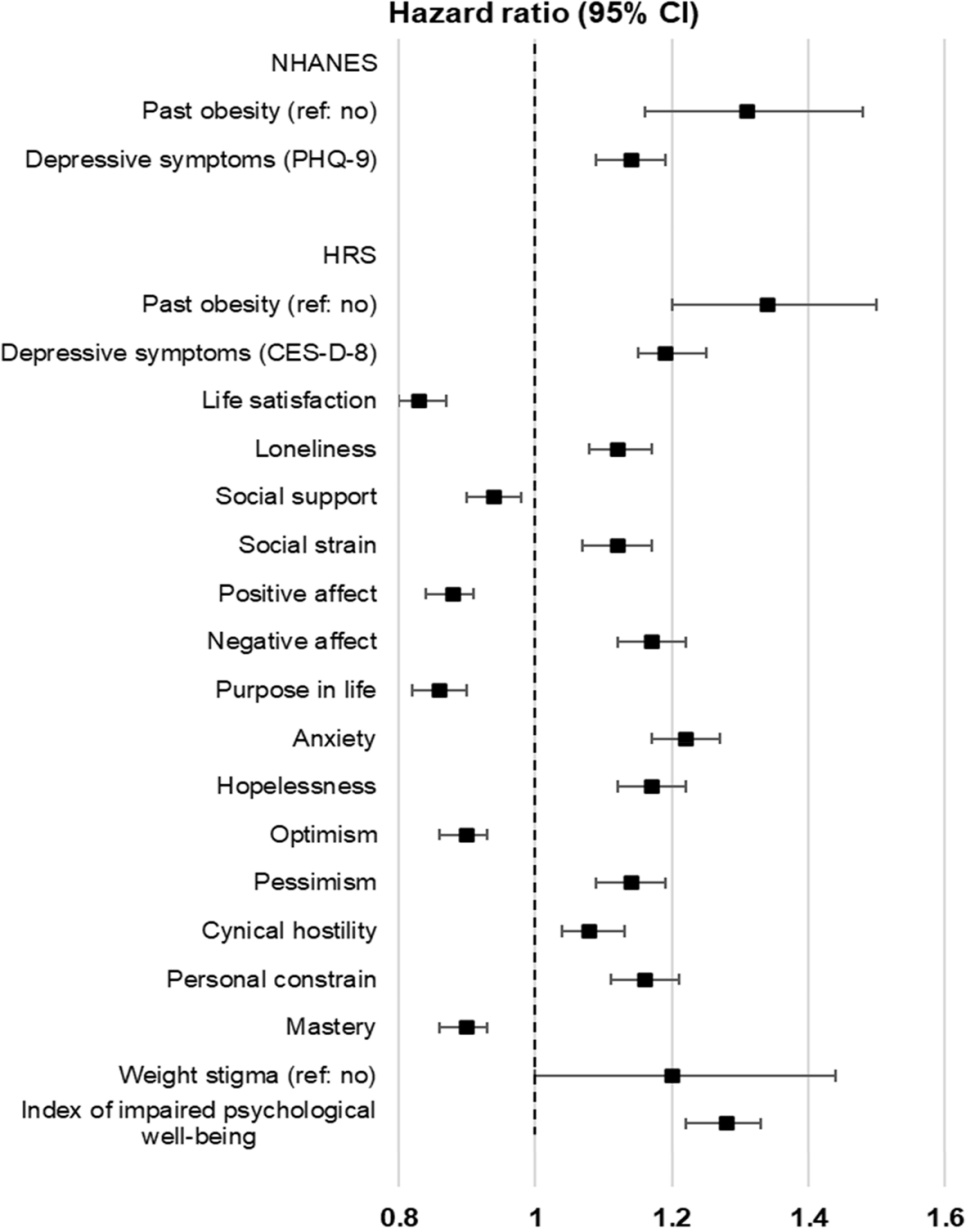
Associations between past obesity, psychological outcomes, and mortality. Associations between past obesity, psychological outcomes, and mortality were examined in separate Cox proportional hazard regression models, adjusting for age, sex, ethnicity, marital status, education, working status, household income (NHANES) or household wealth (HRS), current objective BMI and BMI-squared, and study wave (NHANES). The association between past obesity and mortality are from models not adjusting for psychological outcomes. The associations between each psychological outcome and mortality are from models that do not adjust for past obesity and other psychological outcomes

Cox proportional hazard regression models indicated that participants who had obesity in the past had an increased risk of mortality in both NHANES (HR = 1.31, 95% CI: 1.16, 1.48) and the HRS (HR = 1.34, 95% CI: 1.20, 1.50) compared to those who did not have obesity previously, independently of current weight status (Fig. 2; see Additional file 2: Table S3 for full information). In addition, a 1-SD increase in depressive symptoms was associated with a 14% increased risk of mortality (HR = 1.14, 95% CI: 1.09, 1.19) in NHANES and a 19% increased risk in HRS (HR = 1.19, 95% CI: 1.15, 1.25). Almost all psychological outcomes were associated with mortality in HRS, where a 1-SD increase in the impaired psychological well-being index was associated with a 28% increased risk of mortality (HR = 1.28, 95% CI: 1.22, 1.33).

### Obesity and mortality mediation by psychological outcomes

**Fig. 3:**
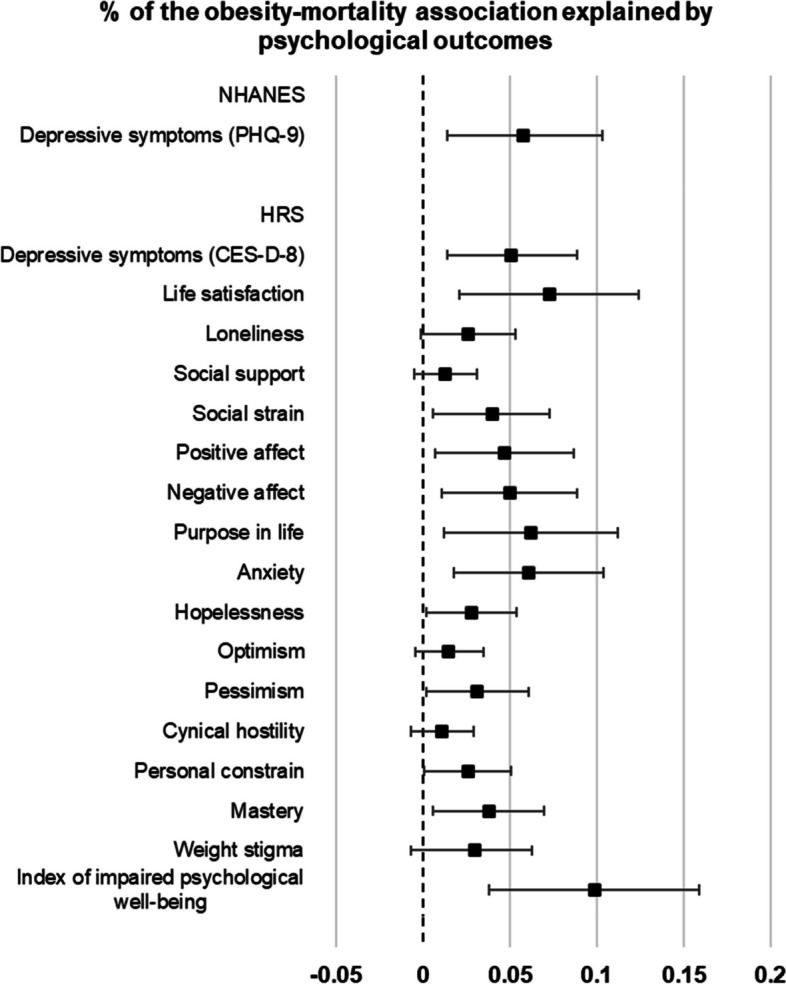
Mediation of the obesity-mortality association by psychological outcomes. Mediation by each psychological outcome was examined in separate single mediation models, adjusting for age, sex, ethnicity, marital status, education, working status, household income (NHANES) or household wealth (HRS), current objective BMI and BMI-squared, and study wave (NHANES). Single mediation models of a psychological outcome do not adjust for other psychological outcomes

Figure 3 presents the overall proportion of the relationship between past obesity and mortality attributed to by each psychological outcome independently, adjusting for sociodemographic covariates and current weight status (full reports of mediation analyses are presented in Additional file 2: Table S4). In NHANES, 6% (95% CI: 0.01, 0.10) of the association between past obesity and mortality was explained by depressive symptoms. Separate single mediation models were conducted for each potential mediating variable in the HRS. Depressive symptoms explained 5% (95% CI: 0.01, 0.09) of the association between past obesity and premature mortality. The mediation analyses indicated several other individual psychological outcomes could explain a statistically significant portion of the association between past obesity (relative to non-obesity) and increased risk of mortality, including life satisfaction, social strain, positive affect, negative affect, purpose in life, anxiety, hopelessness, pessimism, personal constraint, and mastery with the proportion mediated ranging from 3 to 7%. The combined index of impaired psychological well-being explained 10% (95% CI: 0.04, 0.16) of the association between past obesity and mortality.

In HRS, some participants did not have BMI available before baseline and thus their weight history was based on a single measure of BMI at baseline (*n* = 1439). Sensitivity analyses for HRS that excluded these participants were consistent with the main analyses (see Additional file 2: Tables S5 to S7 for full information). We also examined mediation by current depressive symptoms controlling for pre-baseline depressive symptoms and found that the proportion of mediation remained the same (7%; 95% CI: − 0.01, 0.15) but was not statistically significant at *p* < 0.05 (*p* = 0.08), which is likely due to the reduction in sample size from 9868 to 7358 (see Additional file 2: Tables S7 and S8). Additional sensitivity analyses included adjustments for health behaviours and chronic conditions. When the associations between past obesity and current psychological outcomes in Table 2 were adjusted for health behaviours and chronic conditions, most of the associations remained statistically significant (e.g. depressive symptoms, index of impaired psychological well-being) (see Additional file 2: Table S9). In addition, the association between past obesity and current depressive symptoms with pre-baseline depressive symptoms controlled (Additional file 2: Table S2) remained statistically significant following additional adjustments for health behaviours and chronic conditions (see Additional file 2: Table S10). Furthermore, findings from sensitivity analyses in which mediation models presented in Fig. 3 or Additional file 2: Table S4 further adjusted for health behaviours and chronic conditions indicated that the proportion mediated (%) decreased (see Additional file 2: Table S11). Depressive symptoms in both studies were no longer a statistically significant mediator at *p* < 0.05 after this adjustment. However, in HRS, some of the psychological measures remained statistically significant mediators (*p* < 0.05) after both health behaviours and chronic conditions were controlled in the mediation models and effect estimates were similar in size: life satisfaction (6%), anxiety (4%), and the index of impaired psychological well-being (7%).

From all mediation analyses presented in Additional file 2: Tables S4, S7, S8, and S9, “total effect relative risk ratio” or TR for the association between past obesity and risk of mortality from ART models was consistently less than one and statistically significant which indicates that past obesity (vs. without) was associated with shorter disease-free time or shorter survival (i.e. increased risk of mortality). Therefore, findings on the association between past obesity and early mortality were consistent across Cox proportional hazard and AFT models. For all analyses in HRS, we used inverse probability weighting to compensate for the differences in participants’ characteristics associated with being included in the analytical sample. The findings from using inverse probability weighting were similar to the results when baseline weights were used (findings are not presented).

## Discussion

Past obesity was associated with current depressive symptoms across two studies of US adults and a range of psychological outcomes, including life satisfaction, loneliness, social support, social strain, positive affect, negative affect, purpose in life, anxiety, hopelessness, personal constraint, life mastery, weight stigma, and an aggregated index of impaired psychological well-being in older US adults. These associations were independent of current weight status and sociodemographic characteristics, and this apparent psychological burden of past obesity was evident even among participants who no longer had obesity. In HRS, the association between past obesity and current depressive symptoms was also independent of the level of depressive symptoms reported before developing obesity. Collectively, these observational findings suggest that episodes of obesity may be associated with long-term ‘scarring’ consequences on mental health, and there may be a psychological legacy of obesity even among individuals who are no longer medically considered to be living with obesity.

Previous research has focused largely on linking obesity to psychological factors related to well-being, including depression [56], anxiety disorders [57], low life satisfaction [58], loneliness [59], hopelessness [60], and negative affect [61] and at times findings have been contradictory, with some studies even finding obesity as a protective factor of depressive symptoms [62]. Our findings suggest that this research may underestimate the magnitude of the association between obesity and psychological outcomes because the role of past obesity has been overlooked. Given the increased number of people who are developing obesity at early ages [63] and people actively attempting to lose weight [64], a history of obesity is now more common, and the findings of the present study suggest this history of obesity may be associated with the development and/or persistence of depressive symptoms and impaired well-being. Our findings suggest that the psychological burden of obesity may be larger and have longer term consequences than previously assumed.

Although weight loss has been associated with improvements in psychological well-being [65], this improvement may only be partial. Even after weight loss, for example, individuals with a history of obesity may still fear stigmatisation [19]. A qualitative study also suggests that some people who lost weight and were no longer classed as having obesity perceived an enduring threat of discrimination [20]. As such, the potential psychological scarring of past obesity may be explained by previous internalisation of negative stereotypes and beliefs associated with obesity that persists after weight loss and continues to elicit concerns about rejection. Therefore, this also indicates that weight stigma may potentially have a long-lasting impact on health that continues even after weight loss. In addition to weight stigma, living with obesity may be associated with experiencing other forms of trauma. Obesity in early life is associated with adverse experiences (e.g. physical and verbal victimisation, social exclusion) [66, 67] that may continue have long-term psychological consequences even after weight loss.

In this present study, even though we found past obesity was associated with baseline experience of weight stigma, (measured as reports of everyday discrimination), independently of current weight status, weight stigma was not a significant mediator of the association between past obesity and increased risk of mortality. This finding should be interpreted carefully as weight stigma was assessed at baseline only in this study and the experience of weight stigma between baseline and follow-up (i.e. when mortality was recorded) may be more important in explaining health outcomes. Previous research indicated that change in weight stigma over time predicted worsening physiological health, but a single baseline measurement of weight stigma did not predict worsening of health [18].

There are unmeasured other factors that may contribute to the pathways examined in the present research. After previously having obesity experiencing weight cycling (i.e. repeated weight loss and regained) may be associated with worse psychological outcomes [68–70]. In addition, unhealthy weight loss methods can contribute to poorer psychological well-being [71]. Furthermore, the biological consequences of adiposity on the body, such as inflammation [72], can increase the risk of impaired psychological well-being (e.g. depression) [21]. Signs of inflammation may remain in the body after weight loss [22]. Previous obesity may thus be associated with worse long-term well-being through both psychological and biological pathways.

Our findings on the mediation of the association between past obesity and increased risk of mortality through psychological well-being related measures align with evidence for greater risk of premature mortality among individuals with higher levels of psychological distress [73]. Psychological outcomes associated with past obesity may reduce life expectancy directly through physiological changes in the body. For example, depressive symptoms have been found to be associated with inflammation as gauged by C-reactive protein levels [33] and a range of metabolic syndrome components [32]. Health risk behaviours may serve as indirect mechanisms of how the psychological outcomes associated with obesity could increase the risk of premature mortality. Increased negative emotions and impaired psychological well-being levels can undermine self-regulation of health-promoting behaviours to reduce physical activity and increase comfort eating [31]. For this reason, past obesity may also, through worsening psychological well-being, be associated with poor weight management.

In addition to proposed physiological and behavioural mechanisms, mediation by psychological outcomes (e.g. depressive symptoms) may be a sign of having chronic diseases as we found that the proportion of mortality risk mediated by psychological outcomes tended to decrease somewhat when chronic diseases were controlled in the mediation models. Obesity is associated with increased risk of developing chronic conditions that contribute to mortality, such as heart disease and diabetes [74]. These chronic conditions are often correlated with depression [75, 76]. However, it is important to note that some psychological measures (including a summary index of overall well-being) remained significant mediators in all models irrespective of level of adjustment. Furthermore, it is important to note that adjusting for health behaviours and chronic conditions may lead to model over adjustment as both may act as potential mediators of the associations being examined. Less healthy lifestyle behaviours and development of chronic health conditions may result from past obesity related psychological distress and increase risk of mortality. However, the relatively small amount of variance mediated by psychological outcomes may indicate that other pathways through health behaviours, physiological changes, including cardiometabolic pathophysiological processes (e.g. inflammation), and the development of chronic diseases may explain the association between past obesity and increased risk of mortality.

A recent meta-analysis supported the ‘fat and jolly’ hypothesis [62] where overweight and obesity were associated with low depressive symptoms in older adults [77]. However, those studies failed to consider the association between past obesity and psychological well-being in later life. In addition, our approach of investigating the role of past obesity based on the highest BMI in life instead of baseline obesity in being associated with increased risk of premature mortality is supported by recent literature, which suggests that this approach is needed to mitigate possible confounding by illness or reverse causality [34–36]. This source of bias occurs when the risk of premature mortality is predicted by obesity status measured once at baseline, and therefore, the role of previous weight status in initiating disease as well as other pre-existing conditions associated with current weight loss is not accounted for [34]. This bias might explain the unexpected association between obesity and mortality or the ‘obesity paradox’ [78] and, in the context of psychological outcomes, the proposed ‘fat and jolly’ paradox [62].

It will now be important to better understand how and why a history of obesity increases risk of lower psychological well-being and early mortality. For example, although we examined remission of past obesity, some participants with past obesity may have also experienced a history of ‘weight cycling’. Repeated weight loss and regain (weight cycling) may increase risk of mortality [79], though some studies found that the association is not significant following the adjustment for BMI and other risk factors [80, 81]. Participants with past obesity in our studies are best characterised as having recovered from obesity, but we were unable to determine if weight loss occurred recently or participants with past obesity had been recently weight stable. Previous studies examining weight loss trajectories tend to show no association or reduced mortality risk [82, 83], with an exception in older adults [84] who may tend to experience unintentional weight loss due to other health conditions [85]. Our study examining the impact of having ever experienced obesity in the past found consistent findings for psychological outcomes and mortality among a general population sample and older adult sample. These considerations highlight that history of obesity may differ profoundly from one person to another person (dependent on how that history is characterised) and therefore future research examining which characteristics of obesity history contribute to worse mental health and mortality will now be important.

### Strengths, limitations, and implications of the study findings

A strength of the present work was replication and consistency of findings across two studies that used different nationally representative data (NHANES and HRS). A limitation is that both past obesity and psychological outcomes were based on self-reported data. However, self-reported and objectively measured BMI tend to be highly correlated [86]. In addition, while the highest BMI could be obtained in NHANES, the assessment of the highest BMI in HRS participants was based on tracking biennial records available a few years before and up to baseline. Future studies may benefit from examining past obesity based on objective weight history available from younger ages.

It is also important to note relatively similar proportions of participants with past obesity and current obesity in HRS (42.7% vs. 42.4%), although findings were consistent when models were limited to participants with and without current obesity. Furthermore, participants with a history of past obesity (but no obesity at baseline) may have been experiencing illness or temporary weight control which could have reverted to be obesity in the future. Nonetheless, psychological outcomes may in part explain (5–10%) why past obesity increased the risk of early mortality. Future work may benefit from exploring whether mediation by psychological measures is contingent upon some socioeconomic characteristics (e.g. sex, ethnicity, household wealth). Furthermore, because only depressive symptoms measure was available across HRS waves, our analyses testing for potential psychological scarring of past obesity by controlling for pre-baseline psychological outcome were limited to current depressive symptoms.

Findings from the present research indicate that examining past obesity is important to understand the true impact of obesity on health. Our findings suggest that ensuring people with obesity also receive psychological support even after experiencing weight loss is important to avert obesity-related psychological difficulties and physical disease burden. Moreover, in clinical practice, eliciting a history of obesity may help to identify individuals who are more likely to have impaired psychological well-being and increased risk of adverse physical health conditions and mortality.

## Conclusions

Past obesity may be psychologically scarring as we found that past obesity was associated with worse current psychological well-being outcomes and increased risk of mortality independently of current weight status. The psychological legacy of past obesity may partly explain the increased risk of mortality associated with past obesity. Future studies may benefit from using high-quality prospective data with an objective weight history available from younger ages to examine the impact of past obesity on future psychological outcomes and increased risk of mortality.

### Supplementary Information

**Additional file 1.** Information on psychological outcomes.

**Additional file 2: Table S1.** Correlations between psychological outcomes in HRS. **Table S2.** Associations between past obesity and current depressive symptoms adjusting for pre-baseline depressive symptoms in HRS. **Table S3.** Associations between past obesity, psychological outcomes, and mortality. **Table S4.** Mediation by psychological outcomes. **Table S5.** Associations between past obesity and current psychological outcomes excluding participants with only weight history at baseline in HRS. **Table S6.** Associations between past obesity, psychological outcomes, and mortality excluding participants with only weight history in HRS. **Table S7.** Mediation by psychological outcomes excluding participants with only weight history at baseline in HRS. **Table S8.** Mediation by depressive symptoms adjusting for pre-baseline depressive symptoms in HRS. **Table S9.** Associations between past obesity and psychological outcomes adjusting for health behaviours and chronic conditions. **Table S10.** Associations between past obesity and current depressive symptoms adjusting for pre-baseline depressive symptoms, health behaviours, and chronic conditions in HRS. **Table S11.** Mediation by psychological outcomes adjusting for health behaviours and chronic conditions.

## Data Availability

Data are available online (NHANES: https://www.cdc.gov/nchs/nhanes/index.htm; HRS: https://hrs.isr.umich.edu/) with the permission of the data custodians.

## Abbreviations

AFT: Accelerated failure time
BMI: Body mass index
CES-D-8: Center for Epidemiologic Studies Depression Scale (8 items)
CI: Confidence interval
EFA: Exploratory factor analysis
EFTF: Enhanced face-to-face interview
HR: Hazard ratio
HRS: Health and Retirement Study
NDI: National Death Index
NHANES: National Health and Nutrition Examination Survey
OR: Odds ratio
PHQ-9: Patient Health Questionnaire (9 items)
SD: Standard deviation
TR: Time ratio
US: United States of America
