# Supplementary material for: The psychological legacy of past obesity and early mortality: evidence from two longitudinal studies
Source: BMC Med. 2023 Nov 16;21:448. doi: 10.1186/s12916-023-03148-3 (PMC10655273; doi:10.1186/s12916-023-03148-3)
Supplement: Supplementary file 1 — Additional file 1. Information on psychological outcomes. [file 12916_2023_3148_MOESM1_ESM.docx]

Additional file 1: Information on psychological outcomes

**Depressive symptoms (PHQ-9).** In NHANES, depressive symptoms were assessed using a validated nine-item version of the Patient Health Questionnaire (PHQ-9) [1]. Participants were asked how often they have been bothered by the following problems in the last two weeks (e.g., “*Little interest or pleasure in doing things*”, “*Feeling down, depressed or hopeless*”). Their responses on a four-point Likert scale from 0 = “*not at all”* to 3 = “*nearly every day”* were summed to construct a total score ranging from 0 to 27 with a higher score indicating greater depressive symptoms.

**Depressive symptoms** **(CES-D-8).** In HRS, a validated eight-item version of the Centre for Epidemiology Depression Scale (CED-S) was used to evaluate depressive symptoms [2]. Participants’ responses as “*yes*” or “*no*” to questions on whether they felt negative emotions in the past seven days (e.g., “*Did you feel depressed?*”, “*Did you feel everything you did was an effort?*”) were scored as 1 and 0, respectively. Their responses were added together to generate a summary score ranging from 0 to 8, with a higher score indicating greater depressive symptoms.

**Life satisfaction.** Five-item Satisfaction with Life Scale (SWLS) [3] was used to assess life satisfaction. Participants responded to questions related to the overall satisfaction of their life (e.g., “*in most ways my life is close to my ideal*”, “*the conditions of my life are excellent*”) with a seven-point Likert-scale answer option (1 = “*strongly disagree*” to 7 = *“strongly agree”*). The responses of all items were summed to calculate a total score with a possible range of 1 to 35. A higher level of life satisfaction was shown by a higher score.

**Loneliness.** Three-item loneliness UCLA loneliness scale [4], including “*How often do you feel: you lack companionship?*”, “*isolated from others*?”, “*left out*?”, was applied to measure loneliness. Participants’ responses as “*hardly ever or never*,” “*some of the time*,” and “*often*” were scored 1, 2, and 3, respectively, and then responses to all three items were totaled to generate a summary score, following a previous approach [5, 6]. Loneliness score ranged from 1 to 9 with a higher score indicating greater of loneliness.

**Social support.** The level of social support was evaluated as participants’ experiences of positive social support received from different types of relationships (i.e., spouse or partner, children, other immediate family members, and friends). Participants were asked three items (e.g., “*How much do they really understand the way you feel about things?*”, “*How much can you rely on them if you have a serious problem?*”) on a four-point Likert scale response format (1 = “*a lot*” to 4 = “*not at all*”) [6, 7]. Their responses were reverse-coded where a higher value indicates a more positive experience and then averaged for each relationship. The final summary score ranging from 1 to 4 was determined by re-averaging the average social support scores from all the reported relationships.

**Social strain.** Contrary to social support, social strain represents overall negative experiences of social interactions with a spouse or partner, children, other immediate family members, and friends. Participants provided answers (1 = “*a lot*” to 4 = “*not at all*”) to three questions (e.g., “*How much do they criticise you?*”, “*How much do they let you down when you are counting on them?*”) [6, 7]. Similar to the scoring system of social support, responses were also reverse-coded to indicate more negative experiences for a greater score and then averaged for each relationship. An overall score ranging from 1 to 4 was generated by re-averaging the average social strain scores from all the reported relationships.

**Positive affect.** Positive affect domain (13 items) from the Positive and Negative Affect scale (PANAS-X) [8] was used to assess positive affect. This sub-scale documented what participants felt during the last month (e.g., “*During the last 30 days, to what degree did you feel: determined?”, “enthusiastic?*”) with a five-point Likert scale response option (1 = “*not at all*” to 5 = “*very much*”). A total score ranging from 1 to 65 was generated by summing responses from all items, with a higher score indicating greater positive affect.

**Negative affect.** This was assessed using negative affect domain (12 items with responses on a five-point Likert scale from 1 = “*not at all”* to 5 = “*very much*”) from PANAS-X [8]. Participants responded to questions related to their negative emotions during the last month (e.g., “*During the last 30 days, to what degree did you feel: afraid?*”, “*upset?*”). Similar to the scoring system for positive affect, responses from all items were added together, resulting in a total score ranging from 1 to 60. Greater negative affect was indicated by a higher total score.

**Purpose in life.** The purpose in life subscale from the Ryff Measures of Psychological Wellbeing was used [9, 10]. Seven items (e.g., “*I enjoy making plans for the future and working to make them a reality.*”, “*I am an active person in carrying out the plans I set for myself.*”) with a six-point Likert scale answer option (1 = *“strongly disagree”* to 6 = *strongly agree”*) were administrated to participants. A total score ranging from 1 to 42 with a higher score indicating greater purpose in life was created by summing the responses for all items.

**Anxiety.** Five items from Beck Anxiety Inventory (BAI) was used to evaluate anxiety [11]. Participants reported how often they felt the following conditions in the past seven days (e.g., “*I had fear of the worst happening*”, “*I was nervous*”) with four possible options from 1 = “*never*” to 4 = *“most of the time”*. A total score with a possible range of 1 to 20 was generated by summing the responses from all items. A higher level of anxiety was indicated by a higher total score.

**Hopelessness.** Four items with responses on a six-point Likert scale (1 = “*strongly disagree*” to 6 = “*strongly agree*”), of which two items were adapted from each Beck, Weissman [12] and Everson, Kaplan [13] (e.g., “*I feel it is impossible for me to reach the goals that I would like to strive for.*”, “*I don’t expect to get what I really want.”*) were used to quantify hopelessness. Responses from all items on were summed to construct a summary score ranging from 1 to 24 where greater hopelessness was indicated by a higher score.

**Optimism.** Three items with responses on a six-point Likert scale (1=“*strongly disagree*” to 6=“*strongly agree*”) (e.g., “*I’m always optimistic about my future.*”, “*In uncertain times, I usually expect the best.*”) from the Life Orientation Test-Revised (LOT-R) [14] were used to evaluate optimism. Participants’ responses from all items were added together to generate a total score with a possible range from 1 to 18. Greater optimism was indicated by a higher score.

**Pessimism.** The other three items from LOT-R [14] (e.g., “*If something can go wrong for me it will.”, “I hardly ever expect things to go my way*.“) were used to evaluate pessimism. With similar scoring system as optimism, a total score ranging from 1 to 18 was generated by summing the responses from all items.

**Cynical hostility.** Participants responded to five items from the Cook-Medley Hostility Inventory [15, 16] (e.g., “*Most people dislike putting themselves out to help other people.*”, “*Most people will use somewhat unfair means to gain profit or an advantage rather than lose it.*”) with answers on a six-point Likert scale (1 = “*strongly disagree*” to 6 = “*strongly agree*”). A total score ranging from 1 to 30 was created by summing responses from all items, with a higher score indicating greater cynical hostility.

**Personal constraints**. Five items (e.g., “*I often feel helpless in dealing with the problems of life.”*, “*Other people determine most of what I can and cannot do.*”) were used to measure personal constraints [17]. The responses on a six-point Likert scale (1 = “*strongly disagree*” to 6 = “*strongly agree*”) from all items were added together, resulting in a total score with a possible range of 1 to 30. A higher level of personal constraints was shown by a higher score.

**Mastery.** Five items (e.g., “I can do just about anything I really set my mind to.”, “When I really want to do something, I usually find a way to succeed at it.”) on a six-point Likert scale (1 = “strongly disagree” to 6 = “strongly agree”) were used to assess perceived mastery [17]. The responses from all items were summed to create a summary score where greater mastery was indicated by a higher score on scale of 1 to 30.

**Weight stigma.** Weight stigma was assessed as part of forms of discrimination experiences, assessed using the Perceived Everyday Experiences with Discrimination Scale [18] Participants reported how often they experienced on daily basis a set of five discriminatory behaviors (e.g., “*you are treated with less respect or courtesy*”, “*you are threatened or harassed*”) on a six-point Likert scale from “*almost every day*” to “*never*” for different reasons, including their weight. Following previous studies [19, 20], weight stigma was defined if participants had ever reported (from “*almost every day*” to “*less than once a year*”) that their ‘weight’ was the reason for their discrimination experience. Weight stigma was treated as a dichotomous variable (yes; no).

**Developing an index of impaired psychological well-being in the Health and Retirement Study**

Findings from exploratory factor analysis (EFA) indicated 10 out of 16 psychological outcomes (depressive symptoms, life satisfaction, loneliness, positive affect, negative affect, purpose in life, anxiety, hopelessness, pessimism, and personal constraint) were loaded on a single factor (cut-off values of ≥ 0.55 for a good item loading (e.g., as in [21]). These 10 retained psychological outcomes were re-coded to indicate greater impairment by a higher score and then z-score transformed. An index of impaired psychological well-being was developed by restandardising the average z-score values of 10 psychological outcomes to have a mean of zero and standard deviation of one. To examine convergent validity, Spearman’s rank correlation was used to assess the correlations between the index and 10 psychological outcomes and a p-value < 0.05 was considered statistically significant (e.g., as in [22]). As expected, all 10 psychological outcomes were statistically significantly correlated with the index. In addition, the internal consistency was evaluated using Cronbach’s α, and the index had good internal consistency (Cronbach’s α = 0.88).

Item loadings for psychological outcomes

| **Psychological outcomes** | **Item loading** |
| --- | --- |
| **Depressive symptoms** | **0.618** |
| **Life satisfaction** | **0.630** |
| **Loneliness** | **0.668** |
| Social support | 0.454 |
| Social strain | 0.503 |
| **Positive affect** | **0.723** |
| **Negative affect** | **0.704** |
| **Purpose in life** | **0.667** |
| **Anxiety** | **0.653** |
| **Hopelessness** | **0.750** |
| Optimism | 0.505 |
| **Pessimism** | **0.666** |
| Cynical hostility | 0.495 |
| **Personal constrain** | **0.717** |
| Mastery | 0.534 |
| Weight stigma | 0.269 |

Spearman’s rank correlations between the index of impaired psychological well-being and its psychological outcomes

| **Psychological outcomes** | **Correlation coefficient** | **p-value** |
| --- | --- | --- |
| Depressive symptoms | 0.593 | <0.001 |
| Life satisfaction | 0.638 | <0.001 |
| Loneliness | 0.646 | <0.001 |
| Positive affect | 0.743 | <0.001 |
| Negative affect | 0.667 | <0.001 |
| Purpose in life | 0.677 | <0.001 |
| Anxiety | 0.630 | <0.001 |
| Hopelessness | 0.759 | <0.001 |
| Pessimism | 0.683 | <0.001 |
| Personal constrain | 0.740 | <0.001 |

**References**

1. Kroenke K, Spitzer RL, Williams JB: The PHQ-9: validity of a brief depression severity measure. *J Gen Intern Med* 2001, 16(9):606-613.

2. Turvey CL, Wallace RB, Herzog R: A revised CES-D measure of depressive symptoms and a DSM-based measure of major depressive episodes in the elderly. *International psychogeriatrics* 1999, 11(2):139-148.

3. Diener E, Emmons RA, Larsen RJ, Griffin S: The Satisfaction With Life Scale. *Journal of Personality Assessment* 1985, 49(1):71-75.

4. Hughes ME, Waite LJ, Hawkley LC, Cacioppo JT: A Short Scale for Measuring Loneliness in Large Surveys: Results From Two Population-Based Studies. *Research on aging* 2004, 26(6):655-672.

5. Davies K, Maharani A, Chandola T, Todd C, Pendleton N: The longitudinal relationship between loneliness, social isolation, and frailty in older adults in England: a prospective analysis. *The Lancet Healthy Longevity* 2021, 2(2):e70-e77.

6. Putra IGNE, Daly M, Sutin A, Steptoe A, Robinson E: Psychological pathways explaining the prospective association between obesity and physiological dysregulation. *Health Psychology* 2023.

7. Khondoker M, Rafnsson SB, Morris S, Orrell M, Steptoe A: Positive and Negative Experiences of Social Support and Risk of Dementia in Later Life: An Investigation Using the English Longitudinal Study of Ageing. *Journal of Alzheimer's Disease* 2017, 58:99-108.

8. Watson D, Clark LA: The PANAS-X: Manual for the Positive and Negative Affect Schedule - Expanded Form. In: *Positive and Negative Affect Schedule - Expanded Form.* University of Iowa; 1994.

9. Ryff CD: Happiness is everything, or is it? Explorations on the meaning of psychological well-being. *Journal of personality and social psychology* 1989, 57(6):1069-1081.

10. Ryff CD, Keyes CL: The structure of psychological well-being revisited. *J Pers Soc Psychol* 1995, 69(4):719-727.

11. Beck AT, Epstein N, Brown G, Steer RA: An inventory for measuring clinical anxiety: psychometric properties. *J Consult Clin Psychol* 1988, 56(6):893-897.

12. Beck AT, Weissman A, Lester D, Trexler L: The measurement of pessimism: The Hopelessness Scale. *Journal of Consulting and Clinical Psychology* 1974, 42(6):861-865.

13. Everson SA, Kaplan GA, Goldberg DE, Salonen R, Salonen JT: Hopelessness and 4-year progression of carotid atherosclerosis. The Kuopio Ischemic Heart Disease Risk Factor Study. *Arterioscler Thromb Vasc Biol* 1997, 17(8):1490-1495.

14. Scheier MF, Carver CS, Bridges MW: Distinguishing optimism from neuroticism (and trait anxiety, self-mastery, and self-esteem): a reevaluation of the Life Orientation Test. *J Pers Soc Psychol* 1994, 67(6):1063-1078.

15. Cook WW, Medley DM: Proposed hostility and Pharisaic-virtue scales for the MMPI. *Journal of Applied Psychology* 1954, 38(6):414-418.

16. Costa PT, Zonderman AB, McCrae RR, Williams RB: Cynicism and paranoid alienation in the Cook and Medley HO Scale. *Psychosomatic Medicine* 1986, 48(3-4):283-285.

17. Infurna FJ, Mayer A: The effects of constraints and mastery on mental and physical health: Conceptual and methodological considerations. *Psychol Aging* 2015, 30(2):432-448.

18. Williams DR, Yan Y, Jackson JS, Anderson NB: Racial Differences in Physical and Mental Health: Socio-economic Status, Stress and Discrimination. *J Health Psychol* 1997, 2(3):335-351.

19. Daly M, Sutin AR, Robinson E: Perceived Weight Discrimination Mediates the Prospective Association Between Obesity and Physiological Dysregulation: Evidence From a Population-Based Cohort. *Psychol Sci* 2019, 30(7):1030-1039.

20. Robinson E, Sutin A, Daly M: Perceived weight discrimination mediates the prospective relation between obesity and depressive symptoms in U.S. and U.K. adults. *Health Psychol* 2017, 36(2):112-121.

21. Cleare S, Gumley A, Cleare CJ, O’Connor RC: An Investigation of the Factor Structure of the Self-Compassion Scale. *Mindfulness* 2018, 9(2):618-628.

22. Sacre H, Haddad C, Hajj A, Zeenny RM, Akel M, Salameh P: Development and validation of the Socioeconomic Status Composite Scale (SES-C). *BMC Public Health* 2023, 23(1):1619.
