# Supplementary material for: The psychological legacy of past obesity and early mortality: evidence from two longitudinal studies
Source: BMC Med. 2023 Nov 16;21:448. doi: 10.1186/s12916-023-03148-3 (PMC10655273; doi:10.1186/s12916-023-03148-3)
Supplement: Supplementary file 2 — Additional file 2: Table S1. Correlations between psychological outcomes in HRS. Table S2. Associations between past obesity and current depressive symptoms adjusting for pre-baseline depressive symptoms in HRS. Table S3. Associations between past obesity, psychological outcomes, and mortality. Table S4. Mediation by psychological outcomes. Table S5. Associations between past obesity and current psychological outcomes excluding participants with only weight history at baseline in HRS. Table S6. Associations between past obesity, psychological outcomes, and mortality excluding participants with only weight history in HRS. Table S7. Mediation by psychological outcomes excluding participants with only weight history at baseline in HRS. Table S8. Mediation by depressive symptoms adjusting for pre-baseline depressive symptoms in HRS. Table S9. Associations between past obesity and psychological outcomes adjusting for health behaviours and chronic conditions. Table S10. Associations between past obesity and current depressive symptoms adjusting for pre-baseline depressive symptoms, health behaviours, and chronic conditions in HRS. Table S11. Mediation by psychological outcomes adjusting for health behaviours and chronic conditions. [file 12916_2023_3148_MOESM2_ESM.docx]

Additional file 2: Supplementary tables

Table S1. Spearman’s rank correlations between psychological outcomes in the Health and Retirement Study

| **Psychological outcomes** | **1** | **2** | **3** | **4** | **5** | **6** | **7** | **8** | **9** | **10** | **11** | **12** | **13** | **14** | **15** | **16** |
| --- | --- | --- | --- | --- | --- | --- | --- | --- | --- | --- | --- | --- | --- | --- | --- | --- |
| 1. **Depressive symptoms** | **1** |  |  |  |  |  |  |  |  |  |  |  |  |  |  |  |
| 1. **Life satisfaction** | -0.372 | **1** |  |  |  |  |  |  |  |  |  |  |  |  |  |  |
| 1. **Loneliness** | 0.370 | -0.411 | **1** |  |  |  |  |  |  |  |  |  |  |  |  |  |
| 1. **Social support** | -0.141 | 0.282 | -0.303 | **1** |  |  |  |  |  |  |  |  |  |  |  |  |
| 1. **Social strain** | 0.213 | -0.278 | 0.345 | -0.330 | **1** |  |  |  |  |  |  |  |  |  |  |  |
| 1. **Weight stigma** | 0.141 | -0.137 | 0.176 | -0.090 | 0.177 | **1** |  |  |  |  |  |  |  |  |  |  |
| 1. **Positive affect** | -0.385 | 0.456 | -0.403 | 0.300 | -0.204 | -0.129 | **1** |  |  |  |  |  |  |  |  |  |
| 1. **Negative affect** | 0.435 | -0.404 | 0.487 | -0.223 | 0.418 | 0.174 | -0.415 | **1** |  |  |  |  |  |  |  |  |
| 1. **Purpose in life** | -0.308 | 0.337 | -0.351 | 0.249 | -0.171 | -0.150 | 0.568 | -0.356 | **1** |  |  |  |  |  |  |  |
| 1. **Anxiety** | 0.398 | -0.339 | 0.358 | -0.154 | 0.300 | 0.135 | -0.377 | 0.596 | -0.324 | **1** |  |  |  |  |  |  |
| 1. **Hopelessness** | 0.343 | -0.393 | 0.387 | -0.261 | 0.235 | 0.161 | -0.526 | 0.354 | -0.518 | 0.368 | **1** |  |  |  |  |  |
| 1. **Optimism** | -0.231 | 0.383 | -0.259 | 0.221 | -0.165 | -0.108 | 0.437 | -0.312 | 0.367 | -0.272 | -0.397 | **1** |  |  |  |  |
| 1. **Pessimism** | 0.303 | -0.321 | 0.330 | -0.233 | 0.251 | 0.136 | -0.429 | 0.305 | -0.404 | 0.335 | 0.689 | -0.349 | **1** |  |  |  |
| 1. **Cynical hostility** | 0.194 | -0.206 | 0.269 | -0.287 | 0.296 | 0.111 | -0.264 | 0.198 | -0.253 | 0.220 | 0.415 | -0.137 | 0.495 | **1** |  |  |
| 1. **Personal constrain** | 0.352 | -0.378 | 0.414 | -0.236 | 0.275 | 0.138 | -0.509 | 0.448 | -0.484 | 0.413 | 0.596 | -0.317 | 0.494 | 0.343 | **1** |  |
| 1. **Mastery** | -0.237 | 0.389 | -0.299 | 0.219 | -0.180 | -0.106 | 0.471 | -0.361 | 0.407 | -0.320 | -0.412 | 0.388 | -0.274 | -0.135 | -0.493 | **1** |

Table S2. Sensitivity analysis on the associations between past obesity (vs no past obesity) and current depressive symptoms adjusting for pre-baseline measure of depressive symptoms in the Health and Retirement Study

| **Psychological outcomes** | **Past obesity status**  **(obesity vs. non-obesity)** | | | | | |
| --- | --- | --- | --- | --- | --- | --- |
|  | **n** | **Model 1** | | **n** | **Model 2** | |
|  |  | **Estimate (95% CI)** | **p-value** |  | **Estimate (95% CI)** | **p-value** |
| ***Health and Retirement Study (HRS)*** | | | | | | |
| Depressive symptoms (CES-D-8) | 7,358 | 0.11 (0.04, 0.18) | 0.003 | 5,422 | 0.18 (0.04, 0.31) | 0.010 |

n=analytical sample size; CI=confidence interval; ref=reference group; CES-D-8=Center for Epidemiologic Studies Depression Scale (8 items)

Model 1 included all eligible participants and Model 2 included only participants with no obesity at baseline (All the analyses also automatically excluded participants with only weight history at baseline).

Separate regression models were developed for each psychological adjusting for age, sex, ethnicity, marital status, education, working status, household wealth, current objective BMI and BMI-squared, and pre-baseline measure of depressive symptoms.

Depressive symptoms were in the form of z-score (mean=0; SD=1).

Table S3. Associations between past obesity, psychological outcomes, and mortality *(Figure 2 in the main document)*

| **Variables** | **n** | **HR (95% CI)** | **p-value** |
| --- | --- | --- | --- |
| ***National Health and Nutrition Examination Survey (NHANES)*** | | | |
| Past obesity *(ref: non-obesity)* | 29,047 | 1.31 (1.16, 1.48) | <0.001 |
| Depressive symptoms (PHQ-9) | 29,047 | 1.14 (1.09, 1.19) | <0.001 |
| ***Health and Retirement Study (HRS)*** | | | |
| Past obesity *(ref: non-obesity)* | 11,195 | 1.34 (1.20, 1.50) | <0.001 |
| Depressive symptoms (CES-D-8) | 11,223 | 1.19 (1.15, 1.25) | <0.001 |
| Life satisfaction | 11,099 | 0.83 (0.80, 0.87) | <0.001 |
| Loneliness | 11,036 | 1.12 (1.08, 1.17) | <0.001 |
| Social support | 11,179 | 0.94 (0.90, 0.98) | 0.004 |
| Social strain | 11,171 | 1.12 (1.07, 1.17) | <0.001 |
| Positive affect | 11,055 | 0.88 (0.84, 0.91) | <0.001 |
| Negative affect | 11,061 | 1.17 (1.12, 1.22) | <0.001 |
| Purpose in life | 10,993 | 0.86 (0.82, 0.90) | <0.001 |
| Anxiety | 11,051 | 1.22 (1.17, 1.27) | <0.001 |
| Hopelessness | 11,108 | 1.17 (1.12, 1.22) | <0.001 |
| Optimism | 11,047 | 0.90 (0.86, 0.93) | <0.001 |
| Pessimism | 11,037 | 1.14 (1.09, 1.19) | <0.001 |
| Cynical hostility | 10,840 | 1.09 (1.04, 1.13) | <0.001 |
| Personal constrain | 11,090 | 1.16 (1.12, 1.21) | <0.001 |
| Mastery | 11,094 | 0.90 (0.86, 0.93) | <0.001 |
| Weight stigma *(ref: no)* | 11,112 | 1.20 (1.00, 1.43) | 0.054 |
| Index of impaired psychological well-being | 11,169 | 1.28 (1.22, 1.33) | <0.001 |

n=analytical sample size; HR=hazard ratio; CI=confidence interval; ref=reference group; PHQ-9=Patient Health Questionnaire (9 items); CES-D-8=Center for Epidemiologic Studies Depression Scale (8 items)

Separate regression models were developed for obesity and each psychological outcome, adjusting for age, sex, ethnicity, marital status, education, working status, household income (NHANES) or household wealth (HRS), current objective BMI and BMI-squared, and study wave (NHANES).

All continuous psychological outcomes were in the form of z-score (mean=0; SD=1).

Index of impaired psychological well-being was developed by re-standardizing the average standardized scores of 10 psychological outcomes (depressive symptoms, life satisfaction, loneliness, positive affect, negative affect, purpose in life, anxiety, hopelessness, pessimism, and personal constraint).

Table S4. Mediation of the obesity-mortality association by psychological outcomes (“overall proportion due to mediation” in Table S4 presented as “the proportion of the obesity-mortality association explained by psychological outcomes” in Figure 3 in the main document)

***National Health and Nutrition Examination Survey (NHANES)***

| **Estimates** | **Depressive symptoms (PHQ-9)**  **(n=29,047)** | |
| --- | --- | --- |
|  | **Estimate (95% CI)** | **p-value** |
| Total effect relative risk ratio | 0.763 (0.670, 0.856) | <0.001 |
| Proportion of controlled direct effect | 0.887 (0.814, 0.961) | <0.001 |
| Proportion of reference interaction | 0.054 (-0.013, 0.121) | 0.113 |
| Proportion of mediated interaction | -0.053 (-0.108, 0.003) | 0.064 |
| Proportion of pure indirect effect | 0.111 (0.048, 0.174) | 0.001 |
| ***Overall proportion due to mediation*** | ***0.058 (0.014, 0.103)*** | ***0.010*** |
| Overall proportion due to interaction | 0.002 (-0.013, 0.016) | 0.826 |
| Overall proportion eliminated | 0.113 (0.039, 0.186) | 0.003 |

***Health and Retirement Study (HRS)***

| **Estimates** | **Depressive symptoms (CES-D-8)**  **(n=11,195)** | | **Life satisfaction**  **(n=11,072)** | | **Loneliness**  **(n=11,010)** | | **Social support**  **(n=11,152)** | |
| --- | --- | --- | --- | --- | --- | --- | --- | --- |
|  | **Estimate (95% CI)** | **p-value** | **Estimate (95% CI)** | **p-value** | **Estimate (95% CI)** | **p-value** | **Estimate (95% CI)** | **p-value** |
| Total effect relative risk ratio | 0.879 (0.834, 0.923) | <0.001 | 0.885 (0.839, 0.930) | <0.001 | 0.880 (0.835, 0.924) | <0.001 | 0.879 (0.834, 0.924) | <0.001 |
| Proportion of controlled direct effect | 0.922 (0.874, 0.970) | <0.001 | 0.920 (0.868, 0.971) | <0.001 | 0.956 (0.923, 0.988) | <0.001 | 0.986 (0.969, 1.003) | <0.001 |
| Proportion of reference interaction | 0.026 (-0.008, 0.061) | 0.133 | 0.009 (-0.033, 0.050) | 0.686 | 0.019 (-0.009, 0.046) | 0.189 | 0.001 (-0.016, 0.018) | 0.923 |
| Proportion of mediated interaction | -0.023 (-0.051, 0.006) | 0.117 | -0.010 (-0.046, 0.027) | 0.603 | -0.020 (-0.049, 0.010) | 0.189 | -0.001 (-0,019, 0.017) | 0.889 |
| Proportion of pure indirect effect | 0.074 (0.026, 0.122) | 0.002 | 0.082 (0.031, 0.133) | 0.002 | 0.046 (0.011, 0.081) | 0.011 | 0.014 (-0.004, 0.032) | 0.123 |
| ***Overall proportion due to mediation*** | ***0.052 (0.014, 0.090)*** | ***0.007*** | ***0.072 (0.021, 0.123)*** | ***0.006*** | ***0.026 (-0.001, 0.053)*** | ***0.062*** | ***0.013 (-0.005, 0.031)*** | ***0.155*** |
| Overall proportion due to interaction | 0.004 (-0.007, 0.150) | 0.506 | -0.001 (-0.007, 0.004) | 0.681 | -0.001 (-0.009, 0.007) | 0.771 | -0.000 (-0.002, 0.001) | 0.521 |
| Overall proportion eliminated | 0.078 (0.030, 0.126) | 0.001 | 0.081 (0.029, 0.132) | 0.002 | 0.044 (0.012, 0.077) | 0.007 | 0.014 (-0.003, 0.031) | 0.114 |
|  |  | |  | |  | |  | |
| **Estimates** | **Social strain**  **(n=11,144)** | | **Positive affect**  **(n=11,029)** | | **Negative affect**  **(n=11,035)** | | **Purpose in life**  **(n=10,968)** | |
|  | **Estimate (95% CI)** | **p-value** | **Estimate (95% CI)** | **p-value** | **Estimate (95% CI)** | **p-value** | **Estimate (95% CI)** | **p-value** |
| Total effect relative risk ratio | 0.881 (0.836, 0.925) | <0.001 | 0.880 (0.834, 0.925) | <0.001 | 0.881 (0.836, 0.925) | <0.001 | 0.887 (0.840, 0.934) | <0.001 |
| Proportion of controlled direct effect | 0.953 (0.918, 0.988) | <0.001 | 0.936 (0.896, 0.975) | <0.001 | 0.932 (0.887, 0.976) | <0.001 | 0.947 (0.906, 0.989) | <0.001 |
| Proportion of reference interaction | 0.008 (-0.023, 0.038) | 0.622 | 0.018 (-0.014, 0.049) | 0.275 | 0.018 (-0.017, 0.054) | 0.309 | -0.010 (-0.047, 0.026) | 0.579 |
| Proportion of mediated interaction | -0.009 (-0.042, 0.023) | 0.576 | -0.022 (-0.059, 0.015) | 0.250 | -0.019 (-0.053, 0.015) | 0.271 | 0.008 (-0.025, 0.040) | 0.657 |
| Proportion of pure indirect effect | 0.049 (0.012, 0.086) | 0.010 | 0.069 (0.026, 0.112) | 0.002 | 0.069 (0.023, 0.115) | 0.003 | 0.056 (0.016, 0.095) | 0.006 |
| ***Overall proportion due to mediation*** | ***0.039 (0.006, 0.073)*** | ***0.020*** | ***0.047 (0.007, 0.087)*** | ***0.023*** | ***0.050 (0.011, 0.089)*** | ***0.013*** | ***0.063 (0.013, 0.113)*** | ***0.014*** |
| Overall proportion due to interaction | -0.002 (-0.005, 0.002) | 0.366 | -0.004 (-0.120, 0.004) | 0.287 | -0.001 (-0.008, 0.006) | 0.822 | -0.003 (-0.008, 0.002) | 0.275 |
| Overall proportion eliminated | 0.047 (0.012, 0.082) | 0.008 | 0.064 (0.025, 0.104) | 0.001 | 0.068 (0.024, 0.113) | 0.003 | 0.053 (0.011, 0.094) | 0.013 |

| **Estimates** | **Anxiety**  **(n=11,026)** | | **Hopelessness**  **(n=11,082)** | | **Optimism**  **(n=11,020)** | | **Pessimism**  **(n=11,013)** | |
| --- | --- | --- | --- | --- | --- | --- | --- | --- |
|  | **Estimate (95% CI)** | **p-value** | **Estimate (95% CI)** | **p-value** | **Estimate (95% CI)** | **p-value** | **Estimate (95% CI)** | **p-value** |
| Total effect relative risk ratio | 0.876 (0.831, 0.920) | <0.001 | 0.869 (0.824, 0.914) | <0.001 | 0.877 (0.832, 0.922) | <0.001 | 0.877 (0.832, 0.922) | <0.001 |
| Proportion of controlled direct effect | 0.904 (0.851, 0.957) | <0.001 | 0.944 (0.909, 0.980) | <0.001 | 0.971 (0.944, 0.998) | <0.001 | 0.958 (0.926, 0.990) | <0.001 |
| Proportion of reference interaction | 0.035 (-0.004, 0.074) | 0.075 | 0.028 (0.001, 0.055) | 0.046 | 0.013 (-0.008, 0.035) | 0.224 | 0.011 (-0.015, 0.037) | 0.419 |
| Proportion of mediated interaction | -0.032 (-0.066, 0.002) | 0.068 | -0.025 (-0.050, 0.001) | 0.064 | -0.010 (-0.027, 0.008) | 0.260 | -0.011 (-0.034, 0.013) | 0.376 |
| Proportion of pure indirect effect | 0.093 (0.038, 0.147) | 0.001 | 0.052 (0.014, 0.091) | 0.007 | 0.026 (-0.003, 0.054) | 0.074 | 0.042 (0.009, 0.074) | 0.013 |
| ***Overall proportion due to mediation*** | ***0.061 (0.017, 0.104)*** | ***0.006*** | ***0.028 (0.002, 0.054)*** | ***0.033*** | ***0.015 (-0.004, 0.035)*** | ***0.125*** | ***0.031 (0.002, 0.061)*** | ***0.038*** |
| Overall proportion due to interaction | 0.003 (-0.009, 0.015) | 0.614 | 0.003 (-0.008, 0.015) | 0.573 | 0.003 (-0.006, 0.013) | 0.500 | 0.000 (-0.006, 0.006) | 0.943 |
| Overall proportion eliminated | 0.096 (0.043, 0.149) | <0.001 | 0.056 (0.020, 0.091) | 0.002 | 0.029 (0.002, 0.056) | 0.035 | 0.042 (0.010, 0.074) | 0.010 |
|  |  | |  | |  | |  | |
| **Estimates** | **Cynical hostility**  **(n=10,816)** | | **Personal constrain**  **(n=11,064)** | | **Mastery**  **(n=11,067)** | | **Weight stigma**  **(n=11,086)** | |
|  | **Estimate (95% CI)** | **p-value** | **Estimate (95% CI)** | **p-value** | **Estimate (95% CI)** | **p-value** | **Estimate (95% CI)** | **p-value** |
| Total effect relative risk ratio | 0.880 (0.835, 0.925) | <0.001 | 0.870 (0.825, 0.915) | <0.001 | 0.877 (0.831, 0,922) | <0.001 | 0.878 (0.833, 0.923) | <0.001 |
| Proportion due to controlled direct effect | 0.991 (0.971, 1.011) | <0.001 | 0.944 (0.910, 0.979) | <0.001 | 0.973 (0.946, 1.000) | <0.001 | 1.129 (-0.306, 2.561) | 0.123 |
| Proportion due to reference interaction | -0.002 (-0.020, 0.015) | 0.799 | 0.029 (0.002, 0.056) | 0.038 | -0.011 (-0.038, 0.016) | 0.424 | -0.156 (-1.603, 1.291) | 0.833 |
| Proportion due to mediated interaction | 0.001 (-0.010, 0.012) | 0.853 | -0.025 (-0.051, 0.001) | 0.060 | 0.009 (-0.016, 0.035) | 0.476 | 0.009 (-0.073, 0.090) | 0.833 |
| Proportion due to pure indirect effect | 0.010 (-0.006, 0.027) | 0.222 | 0.052 (0.014, 0.090) | 0.007 | 0.029 (0.003, 0,054) | 0.027 | 0.019 (-0.056, 0.095) | 0.615 |
| ***Overall proportion due to mediation*** | ***0.012 (-0.007, 0.030)*** | ***0.223*** | ***0.027 (0.002, 0.052)*** | ***0.036*** | ***0.038 (0.005, 0.070)*** | ***0.022*** | ***0.028 (-0.007, 0.064)*** | ***0.121*** |
| Overall proportion due to interaction | -0.001 (-0.008, 0.005) | 0.716 | 0.004 (-0.008, 0.016) | 0.528 | -0.002 (-0.007, 0.003) | 0.473 | -0.147 (-1.512, 1.218) | 0.833 |
| Overall proportion eliminated | 0.009 (-0.011, 0.029) | 0.364 | 0.056 (0.021, 0.091) | 0.002 | 0.027 (-0.000, 0.054) | 0.053 | -0.128 (-1.561, 1.306) | 0.861 |
|  |  | |  | |  | |  | |
| **Estimates** | **Index of impaired psychological well-being**  **(n=11,143)** | |  |  |  |  |  |  |
|  | **Estimate (95% CI)** | **p-value** |  |  |  |  |  |  |
| Total effect relative risk ratio | 0.876 (0.831, 0.921) | <0.001 |  |  |  |  |  |  |
| Proportion due to controlled direct effect | 0.858 (0.790, 0.926) | <0.001 |  |  |  |  |  |  |
| Proportion due to reference interaction | 0.043 (-0.002, 0.088) | 0.062 |  |  |  |  |  |  |
| Proportion due to mediated interaction | -0.043 (-0.085, -0.000) | 0.048 |  |  |  |  |  |  |
| Proportion due to pure indirect effect | 0.142 (0.072, 0.211) | <0.001 |  |  |  |  |  |  |
| ***Overall proportion due to mediation*** | ***0.099 (0.038, 0.160)*** | ***0.001*** |  |  |  |  |  |  |
| Overall proportion due to interaction | 0.001 (-0.010, 0.012) | 0.914 |  |  |  |  |  |  |
| Overall proportion eliminated | 0.142 (0.074, 0.210) | <0.001 |  |  |  |  |  |  |

n=analytical sample size; CI=confidence intervals; PHQ-9=Patient Health Questionnaire (9 items); CES-D-8=Center for Epidemiologic Studies Depression Scale (8 items)

**Overall proportion due to mediation= proportion** **of the obesity-mortality association mediated (a total of proportions due to mediated interaction and pure indirect effect)**

Overall proportion due to interaction = a total of proportions due to reference and mediated interactions

Overall proportion eliminated = a total proportion due to reference, mediated interactions, and pure indirect effect

The excess relative risks of the total effect and its four components were not presented.

Separate mediation models were developed for each psychological outcome, adjusting for age, sex, ethnicity, marital status, education, working status, household income (NHANES) or household wealth (HRS), current objective BMI and BMI-squared, and study wave (NHANES).

All continuous psychological outcomes were in the form of z-score (mean=0; SD=1).

Index of impaired psychological well-being was developed by re-standardizing the average standardized scores of 10 psychological outcomes (depressive symptoms, life satisfaction, loneliness, positive affect, negative affect, purpose in life, anxiety, hopelessness, pessimism, and personal constraint).

Table S5. Sensitivity analysis on the associations between past obesity (vs no past obesity) and current psychological outcomes excluding participants with only weight history at baseline in the Health and Retirement Study

| **Psychological outcomes** | **Past obesity status**  **(obesity vs. non-obesity)** | | | | | |
| --- | --- | --- | --- | --- | --- | --- |
|  | **n** | **Model 1** | | **n** | **Model 2** | |
|  |  | **Estimate (95% CI)** | **p-value** |  | **Estimate (95% CI)** | **p-value** |
| ***Health and Retirement Study (HRS)*** | | |  | | |  |
| Depressive symptoms (CES-D-8) | 9,868 | 0.15 (0.09, 0.22) | <0.001 | 5,796 | 0.21 (0.09, 0.33) | 0.001 |
| Life satisfaction | 9,756 | -0.13 (-0.20, -0.07) | <0.001 | 5,729 | -0.13 (-0.24, -0.03) | 0.012 |
| Loneliness | 9,698 | 0.12 (0.05, 0.18) | 0.001 | 5,697 | 0.13 (0.02, 0.24) | 0.020 |
| Social support | 9,833 | -0.09 (-0.16, -0.02) | 0.008 | 5,773 | -0.08 (-0.18, 0.03) | 0.147 |
| Social strain | 9,826 | 0.14 (0.08, 0.20) | <0.001 | 5,770 | 0.14 (0.04, 0.25) | 0.006 |
| Positive affect | 9,717 | -0.15 (-0.21, -0.08) | <0.001 | 5,706 | -0.14 (-0.25, -0.02) | 0.017 |
| Negative affect | 9,723 | 0.15 (0.08, 0.22) | <0.001 | 5,711 | 0.22 (0.10, 0.33) | <0.001 |
| Purpose in life | 9,661 | -0.10 (-0.17, -0.04) | 0.002 | 5,654 | -0.06 (-0.16, 0.05) | 0.276 |
| Anxiety | 9,713 | 0.15 (0.08, 0.22) | <0.001 | 5,692 | 0.25 (0.13, 0.37) | <0.001 |
| Hopelessness | 9,768 | 0.08 (0.01, 0.14) | 0.018 | 5,733 | 0.04 (-0.06, 0.14) | 0.463 |
| Optimism | 9,710 | -0.07 (-0.14, 0.00) | 0.051 | 5,700 | -0.06 (-0.18, 0.05) | 0.271 |
| Pessimism | 9,706 | 0.06 (-0.00, 0.13) | 0.062 | 5,697 | 0.04 (-0.06, 0.14) | 0.395 |
| Cynical hostility | 9,539 | 0.04 (-0.02, 0.11) | 0.196 | 5,605 | 0.00 (-0.10, 0.10) | 0.953 |
| Personal constrain | 9,752 | 0.09 (0.02, 0.15) | 0.011 | 5,730 | 0.11 (-0.00, 0.21) | 0.052 |
| Mastery | 9,751 | -0.10 (-0.17, -0.04) | 0.003 | 5,731 | -0.15 (-0.25, -0.04) | 0.007 |
| Weight stigma *(ref: no)* | 9,770 | 3.50 (2.54, 4.80) | <0.001 | 5,726 | 3.45 (2.01, 5.92) | <0.001 |
| Index of impaired psychological well-being | 9,824 | 0.17 (0.10, 0.24) | <0.001 | 5,770 | 0.19 (0.08, 0.31) | 0.001 |

n=analytical sample size; CI=confidence interval; ref=reference group; CES-D-8=Center for Epidemiologic Studies Depression Scale (8 items)

Model 1 included all eligible participants and Model 2 included only participants with no obesity at baseline.

Separate regression models were developed for each psychological adjusting for age, sex, ethnicity, marital status, education, working status, household wealth, and current objective BMI and BMI-squared.

Findings for the adjusted associations between past obesity status and psychological outcomes are presented as regression coefficients (β), except that weight stigma is presented as odds ratio (OR).

All continuous psychological outcomes were in the form of z-score (mean=0; SD=1).

Index of impaired psychological well-being was developed by re-standardizing the average standardized scores of 10 psychological outcomes (depressive symptoms, life satisfaction, loneliness, positive affect, negative affect, purpose in life, anxiety, hopelessness, pessimism, and personal constraint).

Table S6. Sensitivity analysis on the associations between past obesity, psychological outcomes, and mortality excluding participants with only weight history at baseline in the Health and Retirement Study

| **Variables** | **n** | **HR (95% CI)** | **p-value** |
| --- | --- | --- | --- |
| ***Health and Retirement Study (HRS)*** | | |  |
| Past obesity *(ref: non-obesity)* | 9,868 | 1.39 (1.24, 1.56) | <0.001 |
| Depressive symptoms (CES-D-8) | 9,868 | 1.20 (1.15, 1.25) | <0.001 |
| Life satisfaction | 9,756 | 0.83 (0.79, 0.86) | <0.001 |
| Loneliness | 9,698 | 1.13 (1.08, 1.18) | <0.001 |
| Social support | 9,833 | 0.93 (0.90, 0.97) | <0.001 |
| Social strain | 9,826 | 1.13 (1.08, 1.18) | <0.001 |
| Positive affect | 9,717 | 0.87 (0.84, 0.91) | <0.001 |
| Negative affect | 9,723 | 1.17 (1.12, 1.23) | <0.001 |
| Purpose in life | 9,661 | 0.85 (0.82, 0.89) | <0.001 |
| Anxiety | 9,713 | 1.21 (1.16, 1.27) | <0.001 |
| Hopelessness | 9,768 | 1.16 (1.11, 1.21) | <0.001 |
| Optimism | 9,710 | 0.90 (0.86, 0.93) | <0.001 |
| Pessimism | 9,706 | 1.14 (1.09, 1.19) | <0.001 |
| Cynical hostility | 9,539 | 1.10 (1.05, 1.15) | <0.001 |
| Personal constrain | 9,752 | 1.17 (1.12, 1.22) | <0.001 |
| Mastery | 9,751 | 0.89 (0.86, 0.93) | <0.001 |
| Weight stigma *(ref: no)* | 9,770 | 1.21 (1.00, 1.45) | 0.049 |
| Index of impaired psychological well-being | 9,824 | 1.28 (1.22, 1.34) | <0.001 |

n=analytical sample size; HR=hazard ratio; CI=confidence interval; ref=reference group; CES-D-8=Center for Epidemiologic Studies Depression Scale (8 items)

Separate regression models were developed for obesity and each psychological outcome, adjusting for age, sex, ethnicity, marital status, education, working status, household wealth, and current objective BMI and BMI-squared.

All continuous psychological outcomes were in the form of z-score (mean=0; SD=1).

Index of impaired psychological well-being was developed by re-standardizing the average standardized scores of 10 psychological outcomes (depressive symptoms, life satisfaction, loneliness, positive affect, negative affect, purpose in life, anxiety, hopelessness, pessimism, and personal constraint).

Table S7. Sensitivity analysis on mediation of the obesity-mortality association by psychological outcomes excluding participants with only weight history at baseline in the Health and Retirement Study

| **Estimates** | **Depressive symptoms (CES-D-8)**  **(n=9,868)** | | **Life satisfaction**  **(n=9,756)** | | **Loneliness**  **(n=9,698)** | | **Social support**  **(n=9,833)** | | |
| --- | --- | --- | --- | --- | --- | --- | --- | --- | --- |
|  | **Estimate (95% CI)** | **p-value** | **Estimate (95% CI)** | **p-value** | **Estimate (95% CI)** | **p-value** | **Estimate (95% CI)** | | **p-value** |
| Total effect relative risk ratio | 0.865 (0.821, 0.909) | <0.001 | 0.871 (0.826, 0.916) | <0.001 | 0.865 (0.821, 0.910) | <0.001 | 0.865 (0.821, 0.909) | | <0.001 |
| Proportion of controlled direct effect | 0.914 (0.866, 0.961) | <0.001 | 0.916 (0.863, 0.968) | <0.001 | 0.948 (0.914, 0.982) | <0.001 | 0.985 (0.967, 1.003) | | <0.001 |
| Proportion of reference interaction | 0.022 (-0.018, 0.062) | 0.285 | 0.005 (-0.045, 0.056) | 0.833 | 0.027 (-0.006, 0.060) | 0.111 | -0.000 (-0.019, 0.019) | | 0.981 |
| Proportion of mediated interaction | -0.020 (-0.053, 0.013) | 0.243 | -0.006 (-0.044, 0.032) | 0.758 | -0.023 (-0.052, 0.006) | 0.124 | -0.000 (-0,019, 0.019) | | 0.986 |
| Proportion of pure indirect effect | 0.084 (0.038, 0.131) | <0.001 | 0.085 (0.037, 0.133) | 0.001 | 0.048 (0.014, 0.083) | 0.006 | 0.015 (-0.003, 0.033) | | 0.092 |
| ***Overall proportion due to mediation*** | ***0.065 (0.024, 0.106)*** | ***0.002*** | ***0.079 (0.028, 0.130)*** | ***0.003*** | ***0.025 (-0.000, 0.051)*** | ***0.052*** | ***0.015 (-0.003, 0.034)*** | | ***0.108*** |
| Overall proportion due to interaction | 0.002 (-0.007, 0.011) | 0.652 | -0.001 (-0.013, 0.012) | 0.921 | 0.004 (-0.007, 0.014) | 0.476 | -0.000 (-0.001, 0.000) | | 0.155 |
| Overall proportion eliminated | 0.086 (0.039, 0.134) | <0.001 | 0.084 (0.032, 0.137) | 0.002 | 0.052 (0.018, 0.087) | 0.003 | 0.015 (-0.003, 0.033) | | 0.099 |
|  |  | |  | |  | |  | | |
| **Estimates** | **Social strain**  **(n=9,826)** | | **Positive affect**  **(n=9,717)** | | **Negative affect**  **(n=9,723)** | | **Purpose in life**  **(n=9,661)** | | |
|  | **Estimate (95% CI)** | **p-value** | **Estimate (95% CI)** | **p-value** | **Estimate (95% CI)** | **p-value** | **Estimate (95% CI)** | | **p-value** |
| Total effect relative risk ratio | 0.867 (0.823, 0.911) | <0.001 | 0.867 (0.822, 0.912) | <0.001 | 0.867 (0.823, 0.911) | <0.001 | 0.872 (0.826, 0.918) | | <0.001 |
| Proportion of controlled direct effect | 0.952 (0.913, 0.991) | <0.001 | 0.935 (0.898, 0.973) | <0.001 | 0.924 (0.877, 0.971) | <0.001 | 0.948 (0.915, 0.985) | | <0.001 |
| Proportion of reference interaction | 0.002 (-0.039, 0.043) | 0.927 | 0.017 (-0.015, 0.050) | 0.298 | 0.020 (-0.026, 0.066) | 0.394 | -0.002 (-0.032, 0.028) | | 0.918 |
| Proportion of mediated interaction | -0.003 (-0.036, 0.031) | 0.884 | -0.020 (-0.056, 0.016) | 0.269 | -0.018 (-0.055, 0.020) | 0.357 | -0.001 (-0.029, 0.028) | | 0.973 |
| Proportion of pure indirect effect | 0.049 (0.013, 0.084) | 0.007 | 0.068 (0.028, 0.108) | 0.001 | 0.074 (0.030, 0.118) | 0.001 | 0.054 (0.017, 0.091) | | 0.005 |
| ***Overall proportion due to mediation*** | ***0.046 (0.012, 0.080)*** | ***0.008*** | ***0.048 (0.009, 0.086)*** | ***0.015*** | ***0.056 (0.012, 0.097)*** | ***0.007*** | ***0.053 (0.012, 0.095)*** | | ***0.012*** |
| Overall proportion due to interaction | -0.001 (-0.008, 0.007) | 0.885 | -0.003 (-0.009, 0.003) | 0.328 | 0.002 (-0.008, 0.012) | 0.653 | -0.002 (-0.004, -0.000) | | 0.022 |
| Overall proportion eliminated | 0.048 (0.009, 0.087) | 0.016 | 0.065 (0.027, 0.102) | 0.001 | 0.076 (0.029, 0.123) | 0.001 | 0.052 (0.015, 0.089) | | 0.006 |
|  |  |  |  |  |  |  |  | |  |
| **Estimates** | **Anxiety**  **(n=9,713)** | | **Hopelessness**  **(n=9,768)** | | **Optimism**  **(n=9,710)** | | **Pessimism**  **(n=9,706)** | | |
|  | **Estimate (95% CI)** | **p-value** | **Estimate (95% CI)** | **p-value** | **Estimate (95% CI)** | **p-value** | **Estimate (95% CI)** | **p-value** | |
| Total effect relative risk ratio | 0.864 (0.819, 0.908) | <0.001 | 0.855 (0.811, 0.899) | <0.001 | 0.862 (0.818, 0.910) | <0.001 | 0.863 (0.819, 0.907) | <0.001 | |
| Proportion of controlled direct effect | 0.912 (0.864, 0.960) | <0.001 | 0.950 (0.919, 0.981) | <0.001 | 0.972 (0.948, 0.996) | <0.001 | 0.960 (0.930, 0.990) | <0.001 | |
| Proportion of reference interaction | 0.029 (-0.010, 0.068) | 0.148 | 0.028 (0.002, 0.053) | 0.032 | 0.013 (-0.008, 0.033) | 0.221 | 0.012 (-0.017, 0.040) | 0.425 | |
| Proportion of mediated interaction | -0.025 (-0.058, 0.007) | 0.128 | -0.024 (-0.049, 0.000) | 0.054 | -0.011 (-0.029, 0.007) | 0.242 | -0.010 (-0.032, 0.013) | 0.391 | |
| Proportion of pure indirect effect | 0.084 (0.037, 0.132) | 0.001 | 0.047 (0.012, 0.081) | 0.008 | 0.026 (0.001, 0.052) | 0.046 | 0.038 (0.009, 0.068) | 0.011 | |
| ***Overall proportion due to mediation*** | ***0.059 (0.019, 0.100)*** | ***0.004*** | ***0.022 (0.001, 0.044)*** | ***0.042*** | ***0.015 (-0.003, 0.034)*** | ***0.101*** | ***0.028 (0.002, 0.055)*** | ***0.036*** | |
| Overall proportion due to interaction | 0.004 (-0.007, 0.015) | 0.512 | 0.003 (-0.008, 0.151) | 0.573 | 0.002 (-0.006, 0.010) | 0.619 | 0.002 (-0.006, 0.009) | 0.664 | |
| Overall proportion eliminated | 0.088 (0.040, 0.136) | <0.001 | 0.050 (0.019, 0.082) | 0.002 | 0.028 (0.004, 0.052) | 0.022 | 0.040 (0.010, 0.070) | 0.009 | |

| **Estimates** | **Cynical hostility**  **(n=9,539)** | | **Personal constrain**  **(n=9,752)** | | **Mastery**  **(n=9,751)** | | **Weight stigma**  **(n=9,770)** | |
| --- | --- | --- | --- | --- | --- | --- | --- | --- |
|  | **Estimate (95% CI)** | **p-value** | **Estimate (95% CI)** | **p-value** | **Estimate (95% CI)** | **p-value** | **Estimate (95% CI)** | **p-value** |
| Total effect relative risk ratio | 0.866 (0.822, 0.910) | <0.001 | 0.857 (0.813, 0.901) | <0.001 | 0.865 (0.820, 0,909) | <0.001 | 0.864 (0.820, 0.908) | <0.001 |
| Proportion due to controlled direct effect | 0.994 (0.968, 1.020) | <0.001 | 0.944 (0.911, 0.978) | <0.001 | 0.974 (0.947, 1.000) | <0.001 | 1.188 (-0.183, 2.558) | 0.089 |
| Proportion due to reference interaction | -0.008 (-0.036, 0.019) | 0.543 | 0.029 (0.002, 0.055) | 0.035 | -0.014 (-0.040, 0.011) | 0.275 | -0.213 (-1.594, 1.169) | 0.763 |
| Proportion due to mediated interaction | 0.004 (-0.009, 0.016) | 0.595 | -0.024 (-0.050, 0.001) | 0.056 | 0.013 (-0.013, 0.038) | 0.329 | 0.011 (-0.060, 0.081) | 0.763 |
| Proportion due to pure indirect effect | 0.011 (-0.005, 0.027) | 0.175 | 0.052 (0.015, 0.088) | 0.005 | 0.028 (0.004, 0,052) | 0.023 | 0.014 (-0.051, 0.080) | 0.671 |
| ***Overall proportion due to mediation*** | ***0.015 (-0.006, 0.035)*** | ***0.160*** | ***0.027 (0.003, 0.052)*** | ***0.029*** | ***0.041 (0.008, 0.074)*** | ***0.015*** | ***0.025 (-0.004, 0.054)*** | ***0.094*** |
| Overall proportion due to interaction | -0.005 (-0.020, 0.010) | 0.526 | 0.004 (-0.008, 0.016) | 0.477 | -0.002 (-0.008, 0.004) | 0.560 | -0.202 (-1.513 1.109) | 0.763 |
| Overall proportion eliminated | 0.006 (-0.020, 0.032) | 0.645 | 0.056 (0.023, 0.089) | 0.001 | 0.026 (-0.000, 0.053) | 0.051 | -0.188 (-1.558, 1.183) | 0.789 |
|  |  |  |  |  |  |  |  |  |
| **Estimates** | **Index of impaired psychological well-being**  **(n=9,824)** | |  |  |  |  |  |  |
|  | **Estimate (95% CI)** | **p-value** |  |  |  |  |  |  |
| Total effect relative risk ratio | 0.863 (0.819, 0.907) | <0.001 |  |  |  |  |  |  |
| Proportion due to controlled direct effect | 0.855 (0.791, 0.919) | <0.001 |  |  |  |  |  |  |
| Proportion due to reference interaction | 0.046 (-0.003, 0.095) | 0.065 |  |  |  |  |  |  |
| Proportion due to mediated interaction | -0.042 (-0.084, 0.000) | 0.052 |  |  |  |  |  |  |
| Proportion due to pure indirect effect | 0.141 (0.077, 0.205) | <0.001 |  |  |  |  |  |  |
| ***Overall proportion due to mediation*** | ***0.099 (0.042, 0.157)*** | ***0.001*** |  |  |  |  |  |  |
| Overall proportion due to interaction | 0.004 (-0.008, 0.016) | 0.519 |  |  |  |  |  |  |
| Overall proportion eliminated | 0.145 (0.081, 0.209) | <0.001 |  |  |  |  |  |  |

n=analytical sample size; CI=confidence intervals; CES-D-8=Center for Epidemiologic Studies Depression Scale (8 items)

**Overall proportion due to mediation= proportion of the obesity-mortality association mediated (a total of proportions due to mediated interaction and pure indirect effect)**

Overall proportion due to interaction = a total of proportions due to reference and mediated interactions

Overall proportion eliminated = a total proportion due to reference, mediated interactions, and pure indirect effect

The excess relative risks of the total effect and its four components were not presented.

Separate mediation models were developed for each psychological outcome, adjusting for age, sex, ethnicity, marital status, education, working status, household wealth, and current objective BMI and BMI-squared.

All continuous psychological outcomes were in the form of z-score (mean=0; SD=1).

Index of impaired psychological well-being was developed by re-standardizing the average standardized scores of 10 psychological outcomes (depressive symptoms, life satisfaction, loneliness, positive affect, negative affect, purpose in life, anxiety, hopelessness, pessimism, and personal constraint).

Table S8. Sensitivity analysis on mediation of the obesity-mortality association by depressive symptoms adjusting for pre-baseline measure of depressive symptoms in the Health and Retirement Study *(this analysis also excluded participants with only weight history at baseline; see “Sensitivity analyses” in the main document)*

| **Estimates** | **Depressive symptoms (CES-D-8)**  **(n=7,358)** | |
| --- | --- | --- |
|  | **Estimate (95% CI)** | **p-value** |
| Total effect relative risk ratio | 0.912 (0.853, 0.970) | <0.001 |
| Proportion of controlled direct effect | 0.880 (0.783, 0.978) | <0.001 |
| Proportion of reference interaction | 0.047 (-0.035, 0.130) | 0.261 |
| Proportion of mediated interaction | -0.033 (-0.091, 0.025) | 0.268 |
| Proportion of pure indirect effect | 0.105 (0.016, 0.195) | 0.021 |
| ***Overall proportion due to mediation*** | ***0.072 (-0.009, 0.153)*** | ***0.080*** |
| Overall proportion due to interaction | 0.015 (-0.016, 0.045) | 0.343 |
| Overall proportion eliminated | 0.120 (0.023, 0.217) | 0.016 |

n=analytical sample size; CI=confidence intervals; CES-D-8=Center for Epidemiologic Studies Depression Scale (8 items)

**Overall proportion due to mediation= proportion of the obesity-mortality association mediated (a total of proportions due to mediated interaction and pure indirect effect)**

Overall proportion due to interaction = a total of proportions due to reference and mediated interactions

Overall proportion eliminated = a total proportion due to reference, mediated interactions, and pure indirect effect

The excess relative risks of the total effect and its four components were not presented.

Mediation analysis was adjusted for age, sex, ethnicity, marital status, education, working status, household wealth, and current objective BMI and BMI-squared.

Depressive symptoms were in the form of z-score (mean=0; SD=1).

Table S9. Sensitivity analyses on the associations between past obesity (vs no past obesity) and current psychological outcomes (findings presented in Table 2) with additional adjustments for health behaviors and chronic conditions

| **Psychological outcomes** | **Past obesity status**  **(obesity vs. non-obesity)** | | | | | |
| --- | --- | --- | --- | --- | --- | --- |
|  | **n** | **Model 1** | | **n** | **Model 2** | |
|  |  | **Estimate (95% CI)** | **p-value** |  | **Estimate (95% CI)** | **p-value** |
| ***National Health and Nutrition Examination Survey (NHANES)*** | | | | | | |
| Depressive symptoms (PHQ-9) | 27,593 | 0.10 (0.06, 0.14) | <0.001 | 16,675 | 0.12 (0.07, 0.17) | <0.001 |
| ***Health and Retirement Study (HRS)*** | | | | | | |
| Depressive symptoms (CES-D-8) | 11,150 | 0.08 (0.02, 0.14) | 0.014 | 6,451 | 0.17 (0.05, 0.28) | 0.004 |
| Life satisfaction | 11,028 | -0.07 (-0.14, -0.01) | 0.024 | 6,380 | -0.08 (-0.18, 0.02) | 0.129 |
| Loneliness | 10,966 | 0.08 (0.02, 0.15) | 0.012 | 6,344 | 0.11 (0.00, 0.21) | 0.046 |
| Social support | 11,107 | -0.05 (-0.12, 0.01) | 0.123 | 6,423 | -0.05 (-0.15, 0.06) | 0.382 |
| Social strain | 11,099 | 0.10 (0.03, 0.16) | 0.003 | 6,419 | 0.12 (0.02, 0.23) | 0.021 |
| Positive affect | 10,985 | -0.09 (-0.15, -0.03) | 0.006 | 6,352 | -0.07 (-0.02, 0.03) | 0.173 |
| Negative affect | 10,991 | 0.09 (0.02, 0.16) | 0.009 | 6,356 | 0.15 (0.04, 0.26) | 0.010 |
| Purpose in life | 10,924 | -0.06 (-0.13, -0.00) | 0.046 | 6,297 | -0.02 (-0.12, 0.08) | 0.714 |
| Anxiety | 10,983 | 0.10 (0.03, 0.16) | 0.003 | 6,341 | 0.18 (0.06, 0.29) | 0.003 |
| Hopelessness | 11,037 | 0.04 (-0.02, 0.10) | 0.208 | 6,379 | 0.01 (-0.10, 0.11) | 0.926 |
| Optimism | 10,976 | -0.03 (-0.10, 0.04) | 0.359 | 6,345 | -0.04 (-0.14, 0.07) | 0.520 |
| Pessimism | 10,969 | 0.03 (-0.03, 0.09) | 0.392 | 6,341 | -0.00 (-0.10, 0.09) | 0.953 |
| Cynical hostility | 10,774 | 0.00 (-0.06, 0.06) | 0.988 | 6,234 | -0.05 (-0.14, 0.05) | 0.349 |
| Personal constrain | 11,020 | 0.05 (-0.01, 0.11) | 0.119 | 6,376 | 0.07 (-0.03, 0.17) | 0.170 |
| Mastery | 11,023 | -0.07 (-0.14, -0.01) | 0.025 | 6,380 | -0.11 (-0.21, -0.01) | 0.040 |
| Weight stigma *(ref: no)* | 11,043 | 3.31 (2.48, 4.42) | <0.001 | 6,376 | 3.53 (2.11, 5.90) | <0.001 |
| Index of impaired psychological well-being | 11,098 | 0.10 (0.04, 0.16) | 0.001 | 6,419 | 0.12 (0.02, 0.23) | 0.022 |

n=analytical sample size; CI=confidence interval; ref=reference group; PHQ-9=Patient Health Questionnaire (9 items); CES-D-8=Center for Epidemiologic Studies Depression Scale (8 items)

Model 1 included all eligible participants and Model 2 included only participants with no obesity at baseline.

Separate regression models were developed for each psychological adjusting for age, sex, ethnicity, marital status, education, working status, household income (NHANES) or household wealth (HRS), current objective BMI and BMI-squared, study wave (NHANES), health behaviors, and chronic conditions.

In NHANES, physical activity was defined as the number of days per week participants reported an episode of moderate physical activity and the number of days per week participants reported an episode of vigorous physical activity (0-14), smoking was based on whether participants smoke on a daily basis (yes; no), and drinking was ascertained as the number of days of alcohol consumption in the past week (0-7).

In HRS, physical activity was defined as frequency of vigorous physical activity (hardly ever/never; 1-3 times a month; once a week; > 1 a week; every day), smoking was based on whether participants have ever smoked (yes; no), and drinking was determined as the number of days for alcohol consumption in the past week (0-7).

Chronic conditions (hypertension, heart disease, stroke, diabetes, arthritis, cancer) in both NHANES and HRS were assessed based on self-reports on whether the participants have been diagnosed with those health conditions (yes; no) by a health practitioner.

Findings for the adjusted associations between past obesity status and psychological outcomes are presented as regression coefficients (β), except that weight stigma is presented as odds ratio (OR).

All continuous psychological outcomes were in the form of z-score (mean=0; SD=1).

Index of impaired psychological well-being was developed by re-standardizing the average standardized scores of 10 psychological outcomes (depressive symptoms, life satisfaction, loneliness, positive affect, negative affect, purpose in life, anxiety, hopelessness, pessimism, and personal constraint).

Table S10. Sensitivity analysis on the associations between past obesity (vs no past obesity) and current depressive symptoms adjusting for pre-baseline measure of depressive symptoms in the Health and Retirement Study (findings presented in Table S2) with additional adjustments for health behaviors and chronic conditions

| **Psychological outcomes** | **Past obesity status**  **(obesity vs. non-obesity)** | | | | | |
| --- | --- | --- | --- | --- | --- | --- |
|  | **n** | **Model 1** | | **n** | **Model 2** | |
|  |  | **Estimate (95% CI)** | **p-value** |  | **Estimate (95% CI)** | **p-value** |
| ***Health and Retirement Study (HRS)*** | | | | | | |
| Depressive symptoms (CES-D-8) | 7,327 | 0.09 (0.02, 0.17) | 0.012 | 5,396 | 0.16 (0.03, 0.30) | 0.016 |

n=analytical sample size; CI=confidence interval; ref=reference group; CES-D-8=Center for Epidemiologic Studies Depression Scale (8 items)

Model 1 included all eligible participants and Model 2 included only participants with no obesity at baseline (All the analyses also automatically excluded participants with only weight history at baseline).

Separate regression models were developed for each psychological adjusting for age, sex, ethnicity, marital status, education, working status, household wealth, current objective BMI and BMI-squared, pre-baseline measure of depressive symptoms, health behaviors, and chronic conditions.

Physical activity was defined as frequency of vigorous physical activity (hardly ever/never; 1-3 times a month; once a week; > 1 a week; every day), smoking was based on whether participants have ever smoked (yes; no), and drinking was determined as the number of days for alcohol consumption in the past week (0-7).

Chronic conditions (hypertension, heart disease, stroke, diabetes, arthritis, cancer) were assessed based on self-reports on whether the participants have been diagnosed with those health conditions (yes; no) by a health practitioner.

Depressive symptoms were in the form of z-score (mean=0; SD=1).

Table S11. Sensitivity analysis on mediation of the obesity-mortality association by psychological outcomes (“overall proportion due to mediation” in Figure 3 and in Table S4) with additional adjustments for health behaviors and chronic conditions

| **Psychological measures** | **Model 1** | | **Model 2** | | **Model 3** | |
| --- | --- | --- | --- | --- | --- | --- |
|  | **Estimate (95% CI)** | **p-value** | **Estimate (95% CI)** | **p-value** | **Estimate (95% CI)** | **p-value** |
| ***National Health and Nutrition Examination Survey (NHANES)*** | | | | | | |
| Depressive symptoms (PHQ-9) | 0.039 (0.002, 0.076) | 0.040 | 0.038 (-0.006, 0.082) | 0.090 | 0.020 (-0.015, 0.056) | 0.266 |
| ***Health and Retirement Study (HRS)*** | | | | | | |
| Depressive symptoms (CES-D-8) | 0.043 (0.007, 0.079) | 0.020 | 0.040 (0.002, 0.078) | 0.038 | 0.033 (-0.002, 0.069) | 0.066 |
| Life satisfaction | 0.067 (0.013, 0.121) | 0.015 | 0.067 (0.007, 0.124) | 0.028 | 0.061 (0.001, 0.121) | 0.046 |
| Loneliness | 0.022 (-0.004, 0.049) | 0.099 | 0.020 (-0.008, 0.047) | 0.170 | 0.017 (-0.010, 0.044) | 0.215 |
| Social support | 0.008 (-0.007, 0.022) | 0.322 | 0.011 (-0.008, 0.030) | 0.268 | 0.006 (-0.010, 0.021) | 0.483 |
| Social strain | 0.037 (0.003, 0.072) | 0.035 | 0.037 (-0.002, 0.076) | 0.060 | 0.035 (-0.005, 0.074) | 0.085 |
| Positive affect | 0.034 (-0.003, 0.070) | 0.070 | 0.040 (-0.003, 0.084) | 0.067 | 0.030 (-0.009, 0.069) | 0.134 |
| Negative affect | 0.046 (0.006, 0.086) | 0.025 | 0.044 (0.001, 0.088) | 0.047 | 0.040 (-0.003, 0.084) | 0.069 |
| Purpose in life | 0.052 (0.003, 0.101) | 0.037 | 0.055 (0.005, 0.097) | 0.049 | 0.046 (-0.006, 0.100) | 0.084 |
| Anxiety | 0.057 (0.013, 0.101) | 0.012 | 0.045 (0.004, 0.087) | 0.033 | 0.043 (0.001, 0.086) | 0.045 |
| Hopelessness | 0.021 (-0.002, 0.044) | 0.074 | 0.023 (-0.004, 0.049) | 0.091 | 0.017 (-0.006, 0.040) | 0.146 |
| Optimism | 0.014 (-0.006, 0.033) | 0.178 | 0.012 (-0.008, 0.033) | 0.242 | 0.011 (-0.010, 0.032) | 0.298 |
| Pessimism | 0.025 (-0.003, 0.052) | 0.075 | 0.022 (-0.005, 0.050) | 0.114 | 0.018 (-0.007, 0.043) | 0.166 |
| Cynical hostility | 0.008 (-0.009, 0.024) | 0.360 | 0.006 (-0.014, 0.026) | 0.536 | 0.003 (-0.014, 0.021) | 0.715 |
| Personal constrain | 0.023 (-0.002, 0.047) | 0.071 | 0.026 (-0.003, 0.055) | 0.078 | 0.023 (-0.005, 0.050) | 0.115 |
| Mastery | 0.035 (0.001, 0.069) | 0.045 | 0.035 (-0.002, 0.072) | 0.063 | 0.033 (-0.006, 0.071) | 0.093 |
| Weight stigma *(ref: no)* | 0.028 (-0.014, 0.069) | 0.193 | 0.029 (-0.018, 0.075) | 0.232 | 0.024 (-0.025, 0.073) | 0.332 |
| Index of impaired psychological well-being | 0.086 (0.026, 0.146) | 0.005 | 0.085 (0.020, 0.151) | 0.011 | 0.074 (0.011, 0.138) | 0.021 |

n=analytical sample size; CI=confidence intervals; CES-D-8=Center for Epidemiologic Studies Depression Scale (8 items)

**Overall proportion due to mediation= proportion of the obesity-mortality association mediated (a total of proportions due to mediated interaction and pure indirect effect)**

Separate mediation models were developed for each psychological outcome, adjusting for age, sex, ethnicity, marital status, education, working status, household income (NHANES) or household wealth (HRS), current objective BMI and BMI-squared, and study wave (NHANES), and with additional adjustments for the following models:

Model 1: + adjustments for categorial variables of health behaviors (physical activity, smoking, drinking)

Model 2: + adjustments for binary variables (yes; no) of chronic conditions (hypertension, heart problems, stroke, diabetes, arthritis, cancer)

Model 3: + adjustments for both health behaviors and chronic conditions.

In NHANES, physical activity was defined as the number of days per week participants reported an episode of moderate physical activity and the number of days per week participants reported an episode of vigorous physical activity (0-14), smoking was based on whether participants smoke on a daily basis (yes; no), and drinking was ascertained as the number of days of alcohol consumption in the past week (0-7).

In HRS, physical activity was defined as frequency of vigorous physical activity (hardly ever/never; 1-3 times a month; once a week; > 1 a week; every day), smoking was based on whether participants have ever smoked (yes; no), and drinking was determined as the number of days for alcohol consumption in the past week (0-7).

Chronic conditions in both NHANES and HRS were assessed based on self-reports on whether the participants have been diagnosed with those health conditions (yes; no) by a health practitioner.

All continuous psychological outcomes were in the form of z-score (mean=0; SD=1).

Index of impaired psychological well-being was developed by re-standardizing the average standardized scores of 10 psychological outcomes (depressive symptoms, life satisfaction, loneliness, positive affect, negative affect, purpose in life, anxiety, hopelessness, pessimism, and personal constraint).
